# A Review of Solar Thermochemical CO_2_ Splitting Using Ceria-Based Ceramics With Designed Morphologies and Microstructures

**DOI:** 10.3389/fchem.2019.00601

**Published:** 2019-09-04

**Authors:** Robert C. Pullar, Rui M. Novais, Ana P. F. Caetano, Maria Alexandra Barreiros, Stéphane Abanades, Fernando A. Costa Oliveira

**Affiliations:** ^1^Department of Materials and Ceramic Engineering, CICECO—Aveiro Institute of Materials, University of Aveiro, Aveiro, Portugal; ^2^Renewable Energy and Energy System Integration Unit, LNEG—Laboratório Nacional de Energia e Geologia I.P., LEN—Laboratório de Energia, Lisbon, Portugal; ^3^Processes, Materials, and Solar Energy Laboratory (PROMES-CNRS), Perpignan, France

**Keywords:** CO_2_ splitting, concentrating solar technology, ceria CeO_2_, thermochemical cycle, microstructure, RPC reticulated porous ceramics, solar fuels, biomorphic/biomimetic

## Abstract

This review explores the advances in the synthesis of ceria materials with specific morphologies or porous macro- and microstructures for the solar-driven production of carbon monoxide (CO) from carbon dioxide (CO_2_). As the demand for renewable energy and fuels continues to grow, there is a great deal of interest in solar thermochemical fuel production (STFP), with the use of concentrated solar light to power the splitting of carbon dioxide. This can be achieved in a two-step cycle, involving the reduction of CeO_2_ at high temperatures, followed by oxidation at lower temperatures with CO_2_, splitting it to produce CO, driven by concentrated solar radiation obtained with concentrating solar technologies (CST) to provide the high reaction temperatures of typically up to 1,500°C. Since cerium oxide was first explored as a solar-driven redox material in 2006, and to specifically split CO_2_ in 2010, there has been an increasing interest in this material. The solar-to-fuel conversion efficiency is influenced by the material composition itself, but also by the material morphology that mostly determines the available surface area for solid/gas reactions (the material oxidation mechanism is mainly governed by surface reaction). The diffusion length and specific surface area affect, respectively, the reduction and oxidation steps. They both depend on the reactive material morphology that also substantially affects the reaction kinetics and heat and mass transport in the material. Accordingly, the main relevant options for materials shaping are summarized. We explore the effects of microstructure and porosity, and the exploitation of designed structures such as fibers, 3-DOM (three-dimensionally ordered macroporous) materials, reticulated and replicated foams, and the new area of biomimetic/biomorphous porous ceria redox materials produced from natural and sustainable templates such as wood or cork, also known as ecoceramics.

## Solar Thermochemical Fuel Production

Renewable solar fuels can be generated from the sun, water, and carbon dioxide using existing concentrating solar technology (CST). A thermochemical redox process, driven by concentrated solar radiation as the source of the high temperatures needed, is the basis of much of this work. This allows us to convert sunlight directly to chemical fuels, such as in the splitting of CO_2_, a major by-product of virtually all combustion, agricultural and industrial processes, to produce CO, which can then be used to create renewable synthetic hydrocarbon fuels (Graves et al., 2011; Lanzafame et al., 2017). This usually operates via a two-step redox cycle, consisting of an initial solar-driven high temperature reduction of a material (such as ceria), and its subsequent oxidation at a lower temperature by CO_2_ to create CO.

The two-step redox cycles process appears simple at first: a concentrated and focused beam of sunlight heats the reactive material, in this case ceria up to 1,400°C or more, driving its endothermic reduction and releasing oxygen as a result. The reduced ceria is then cooled (non-solar) to 1,000°C or below (known as a temperature-swing cycle), and re-oxidized under a flow of carbon dioxide, creating carbon monoxide. One of the most critical aspects is solar-to-fuel energy conversion efficiency, defined as the ratio of the heating value of the CO produced to the solar energy input, which depends upon the redox material and the solar thermochemical reactor design (Siegel et al., 2013; Lange et al., 2015; Koepf et al., 2017). The morphology of the ceria redox material can also have a major effect on this. Indeed, reaction kinetics is tied directly to reaction extent, which, in turn, is improved through the selection of high surface area materials.

**Figure 1:**
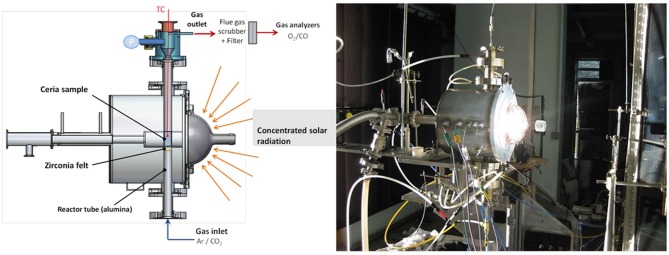
Schematic and photograph of the solar reactor mounted on a medium size solar furnace (MSSF) used in PROMES-CNRS for experiments on two-step solar-driven thermochemical splitting of CO_2_.

There are several major concentrated solar energy facilities throughout the world, which operate either with high flux solar simulators (HFSS) with furnaces heated by simulated solar light, or high flux solar furnaces (HSSF) utilizing actual (not simulated) solar energy, which can be used to study these processes and to carry out tests on candidate materials. The largest solar furnaces, such as that used by the authors at PROMES-CNRS in Odeillo, France, can offer a thermal power of 1 MW or more. They use an array of heliostats (two-axis sun-tracking parabolic or flat mirrors) that focus the sun's rays onto a second, larger parabolic mirror, which is used to concentrate the sunlight to over 16,000 times. The amount (flux) of this light allowed into the furnace through an aperture can control the temperature inside it, and this concentrated solar energy can be used to drive the endothermic reduction step of the thermochemical redox reaction of ceria. The oxidation step is performed during cooling without exposure to concentrated sunlight as an energy source (cut off via the use of shutters). This overview will focus on the solar thermochemical splitting of CO_2_ alone, as opposed to CO_2_/H_2_O splitting to produce syngas (CO + H_2_) mixtures. Much of the research on such materials is carried out on thermogravimetric analysis (TGA) equipment or in electric or infrared furnaces, but many of the papers examined in this review use simulated concentrated solar light to power the reaction. It will be stated where solar powered splitting experiments were carried out using actual solar energy, instead of simulated solar light. The actual concentrated solar light reactor used by the authors at PROMES is shown in Figure 1.

Identification of proper reactive materials with both stable redox properties during cycling and optimal shaping and morphology for integration in solar reactors is required. After a brief discussion of the use of ceria to thermochemically split CO_2_, we look at the effects of microstructure and porosity on this process, and the exploitation of designed structures such as fibers, 3-DOM (three-dimensionally ordered macroporous) materials, reticulated and replicated foams, and the new area of biomimetic/biomorphous porous ceria redox materials produced from natural and sustainable templates such as wood or cork (also known as ecoceramics), in approximately the chronological order in which such types of bulk 3D materials have been developed.

## Ceria for CO_2_ Splitting

Cerium dioxide (ceria) is a versatile reducible oxide with a wide range of applications in catalysis. Pure stoichiometric CeO_2_ has the calcium fluoride (fluorite) type of structure, which is known to tolerate a considerable reduction without phase change, especially at elevated temperatures. Cerium also forms cerium (III) oxide, Ce_2_O_3_, which is unstable and will oxidize to cerium (IV) oxide. Ceria undergoes partial reduction at high temperatures in low pO_2_ (10^−5^ atm) atmospheres, and can support a large extent of oxygen deficiency without change of crystal phase (Carrillo and Scheffe, 2017). CeO_2_ has an unusually high entropy change associated with oxygen exchange compared to other non-stoichiometric redox materials (Lorentzou et al., 2015; Takacs et al., 2016, 2017), resulting in reduced temperature swings between the reduction and oxidation steps (Siegel et al., 2013). Ceria also has rapid reaction kinetics and oxygen diffusion rates (Ackermann et al., 2014a), is thermally stable and relatively resistant to sintering even at high temperatures due to its high melting point (~2,400°C), and keeps its cubic fluorite structure during thermochemical cycling over the range of operating temperatures used (Mogensen et al., 2000). In practice, CeO_2_ requires high operating temperatures (i.e., exceeding 1,773 K, depending on the oxygen partial pressure) in order to achieve the highest efficiencies, owing to its relatively high enthalpy change during oxygen exchange. Thus, CeO_2_ durability issues, such as volatility, chemical, and physical compatibility with other reactor components (e.g., solid/solid reaction, thermal expansion, etc.), and physical degradation associated with thermal cycling need to be considered.

The solar-to-fuel energy conversion efficiency depends upon the oxygen storage and release capacity of ceria, its radiative heat absorptivity, and the kinetics of its reaction with CO_2_. From determinations of diffusion coefficients of pure ceria pellets below 1,500°C, <10 s reduction reaction times were estimated for bulk diffusion length on a scale of ≤ 0.4 mm (Ackermann et al., 2014a). Due to these rapid reaction rates and small diffusion lengths, reduction in solar reactors is probably limited by the rate of heat transfer, rather than chemical kinetics. However, the story is different for oxidation in CO_2_, in which the reaction rate is primarily dictated by the chemical kinetics, not heating rate, and is very dependent upon microstructure and available surface area, as well as chemical composition (Ackermann et al., 2014a; Takacs et al., 2017).

Ceria was first investigated as a material specifically for solar thermochemical CO_2_ splitting in 2010 by Chueh et al. (2010) and Chueh and Haile (2010) and Haussener and Steinfeld (2010, 2012), although it had already been studied for thermochemical water splitting in 2006 by Abanades and Flamant (2006). Since then, many studies have investigated ceria redox materials, as they showed higher oxygen ion mobility and rapid fuel production kinetics compared to ferrites and other non-volatile metal oxides (Abanades and Flamant, 2006; Chueh and Haile, 2010; Bhosale et al., 2019).

The two step CO_2_ splitting cycle of CeO_2_ is based on:

1. The solar thermal reduction of CeO_2_ (endothermic, high temperature) at low oxygen partial pressure in a neutral (typically Ar or N_2_) atmosphere, to create oxygen-deficient non-stoichiometric ceria (CeO_2−δ_, where δ is the degree of oxygen deficiency) via the formation of oxygen vacancies and the subsequent release of O_2_ gas; and
2. The non-solar oxidation of CeO_2−δ_ back to CeO_2_ (exothermic, lower temperature), that will take oxygen from CO_2_ as the temperature is decreased with CO_2_ present, releasing CO gas and re-incorporating some oxygen into the ceria lattice.

**Figure 2:**
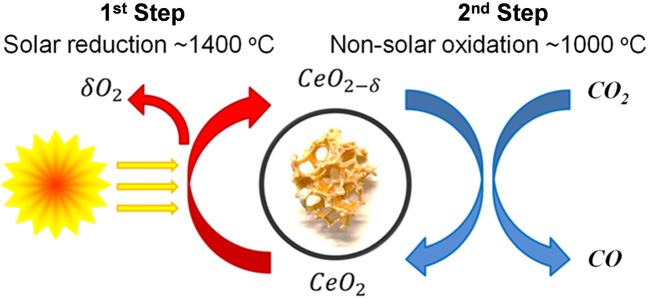
The two-step thermochemical redox process for the spitting of CO_2_ using ceria.

This redox process is depicted in Figure 2. The fuel production yield is dependent on the degree of non-stoichiometry (δ), and is determined by temperature and oxygen partial pressure. Ceria can accommodate quite high amounts of oxygen non-stoichiometry, and can support high levels of oxygen storage/loss and mobility while maintaining the crystallographic fluorite structure (Mogensen et al., 2000). As 1 molecule of O_2_ from the reduction step should produce 2 molecules of CO in the oxidation step, the ideal ratio of O_2_:CO production would equal 2.

**Figure 3:**
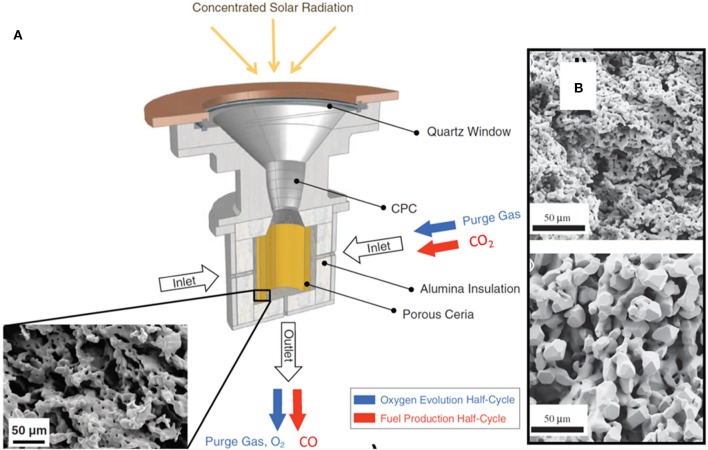
The first report, by Chueh et al. to use the redox reaction of CeO_2_ powered by concentrated solar energy to split CO_2_, using porous, monolithic ceria. **(A)** Schematic of the first solar reactor used for the two-step, solar-driven thermochemical splitting of CO_2_ with ceria. It consisted of a thermally insulated cavity receiver containing an 80% porous monolithic ceria cylinder. The inset shows a SEM image of the porous ceria tube after 23 cycles. Blue arrows indicate ceria reduction, and red arrows indicate oxidation. **(B)** SEM images of the porous CeO_2_ monolith (sintered for 3 h at 1500°C) before and after 500 thermochemical cycles between 800 and 1,500°C. **(A)** Reproduced from https://newatlas.com/breakthrough-solar-reactor-makes-fuel-from-sunlight/17377/, **(B)** From Chueh and Haile (2010), used with permission.

In their first paper, Chueh et al. actually used a real concentrated solar energy furnace (C = 1,500 suns, 1.9 kW) to split CO_2_, with reduction at 1,581–1,624°C for ~50 min, and rapid oxidation at 800°C for ~4 min (see Figure 3A; Chueh et al., 2010). They used porous, monolithic ceria, assembled from quarter-circular-arc pieces to form a fairly large cylinder (35 mm diameter, 102 mm in height, 325 g, 80% porosity as fabricated), and carried out 4 consecutive cycles, in which reduction was seen to begin at 900°C. The peak value of O_2_ production was 34 ml min^−1^ (= 0.105 ml min^−1^ g^−1^) and a very high peak value of CO production rate of 1,500 ml min^−1^ (= 4.6 ml min^−1^ g^−1^) was achieved during the first cycle, although values did diminish with subsequent cycles (by ~30% after 5 cycles), at least partly due to the decrease in reduction temperature (Chueh et al., 2010). After a stabilization of 5 cycles, production remained constant for a further 23–100 cycles, and even up to 500 cycles by TGA, but with considerable grain growth in the ceria from 5 to 15 μm (Figure 3B; Chueh and Haile, 2010). This is related to a strong decrease in reaction rates as a result of sintering. In general, structures with nm-range pores are more prone to sinter, thereby losing a substantial amount of SSA over the initial cycles. This indicates that oxidation of ceria with CO_2_ is a surface-limited process, as the oxidation rate is strongly linked to the SSA (Chueh and Haile, 2010). An overall solar-to-fuel energy conversion of only 0.4% was achieved, but they predicted that values as high as 16 to 19% should be attainable, even without sensible heat recovery (Chueh et al., 2010).

**Figure 4:**
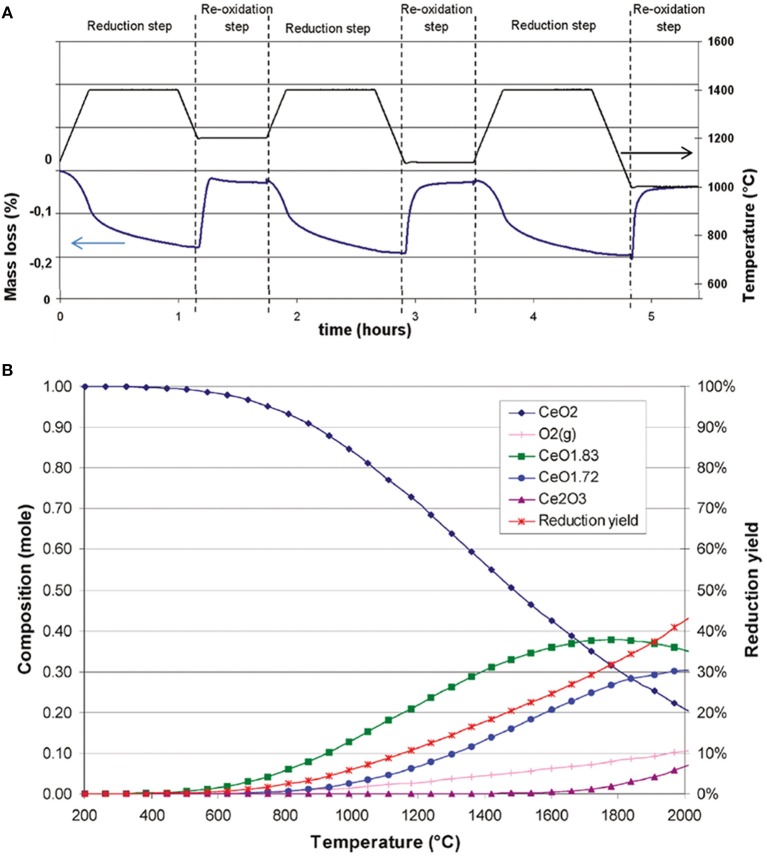
Reactivity of ceria during the CO_2_ splitting cycle investigated by TGA. **(A)** TG analysis of pure CeO_2_, synthesized from coprecipitation of hydroxides, during three consecutive CO_2_ splitting cycles. **(B)** Equilibrium phase composition and theoretical reduction yield predicted by thermodynamics for reduction of the CeO_2_ system under 1 bar of Ar. Adapted with permission from Le Gal et al. (2011). Copyright 2011 American Chemical Society.

The reactivity of ceria during the CO_2_ splitting cycle was investigated by TGA, using a pure ceria synthesized by co-precipitation of hydroxides, and subsequently calcined at 800°C (Le Gal et al., 2011). Three successive thermochemical cycles were carried out, with the reduction step at 1,400°C under Ar, and three oxidation steps under CO_2_ at 1,200, 1,100, and 1,000°C, respectively, as shown in Figure 4A. The lower the temperature, the higher the resulting CO production yield. This was explained by thermodynamic limitation—the exothermic reaction was not favored by a temperature increase. The shape of the first re-oxidation peak at 1,200°C showed that equilibrium occurred between oxidation and reduction, because the mass increased rapidly as soon as CO_2_ was injected, but then decreased slightly due to reduction occurring even during the CO_2_ injection. This showed that at a temperature of 1,200°C oxidation competed with reduction, and the optimal temperature here for the exothermic step was 1,000°C. The theoretical maximum amount of oxygen that can be released per gram of ceria (corresponding to the total reduction of Ce^4+^ to Ce^3+^) is 1.45 mmol (4.65% mass loss), and the maximum possible CO production would be 2.9 mmol g^−1^ (~64.9 ml g^−1^) (Rhodes et al., 2015). During the reduction step at 1,400°C, the average amount of oxygen released for the three cycles was 53 ± 4 μmol g^−1^ (~1.19 ml g^−1^), corresponding to an average reduction yield of 3.7%, a quite low value. The three oxidation steps at 1,200, 1,100, and 1,000°C gave yields of 98, 102 and 105 μmol g^−1^ of CO (~2.28 ml g^−1^), respectively, so there was only a slight increase in yield with decreasing oxidation temperature. This corresponds to about 3.45% of the maximum theoretical CO yield, if all the ceria was reduced to Ce^3+^, and then totally re-oxidized, and such results are typical for standard micron-scale ceria powders. The amount of oxygen released was relatively stable during cycling, and the fact that the oxidation step did not show any diffusion limitation suggested that reduction was restricted to the particle surface, consistent with the low reduction yield, and that oxidation was also a surface-controlled process. A simulation predicted the creation of non-stoichiometric phases of ceria (CeO_1.83_ and CeO_1.72_) at around 500 and 800°C, respectively, while the proper Ce_2_O_3_ phase would begin to form at about 1,600°C (Figure 4B). The simulated theoretical reduction yield increased with temperature to 42% at 2,000°C, but as the model indicated a value of 18% at 1,400°C (much greater than that observed experimentally), kinetic limitations were deemed to be the controlling factors during the reduction of ceria (Le Gal et al., 2011).

Another TGA study on pure ceria (1 μm particles, 60% density due to use of carbon pore forming agent, reduction at 1,500°C for 100 min, oxidation at 1,000°C for 175 min, two cycles) produced 128 and 125 μmol g^−1^ of O_2_ (~2.87 ml g^−1^, 8.83 and 8.61% reduction yield) and 256 and 231 μmol g^−1^ of CO (~5.17–5.73 ml g^−1^) (Bonk et al., 2015). After 100 min at 1,500°C, the mass loss of CeO_2_ was ~0.40%, and when oxidized with CO_2_ at 1,000°C, it reached 90% of its initial oxidation state (a mass gain of 0.36%) after 2 min, oxidation proceeding to completion after a total of 9 min. During the 2nd redox cycle, the mass lost during reduction was 0.4%, while the mass gained during the following re-oxidation was again only 0.37%, indicating a slightly incomplete oxidation process. They also showed that ceria sintering begins at 955°C and that the peak sintering temperature was 1,390°C, and grains reached 60 μm after 5 h at 1,600°C.

**Figure 5:**
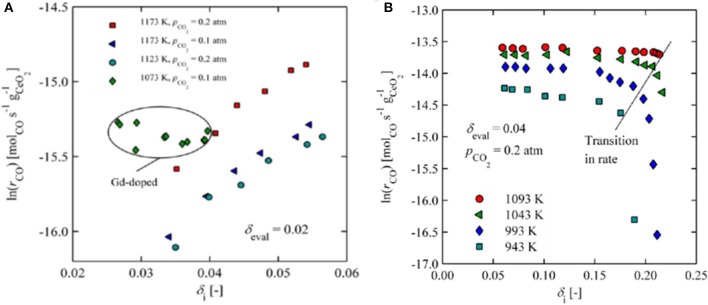
Effects of oxygen non-stoichiometry obtained during reduction on the oxidation kinetics of CeO_2_. **(A)** CO production rates as a function of increasing initial non-stoichiometry (δ < 0.6) for thermally reduced ceria (values shown for various oxidation temperatures and CO_2_ concentrations, and also for 10 mol% Gd-doped ceria). **(B)** CO production rates as a function of increasing initial non-stoichiometry (δ = 0.6–0.22) for ceria chemically reduced under H_2_ (values shown for various oxidation temperatures). Adapted with permission from Ackermann et al. (2015). Copyright 2015 American Chemical Society. Further permissions related to the material excerpted should be directed to the ACS.

The oxygen non-stoichiometry obtained during reduction also strongly affects the oxidation kinetics of CeO_2_. CeO_2_ becomes increasingly reduced to CeO_2−δ_ with increasing temperature, reaching δ up to ~0.03 at 1,500°C even with some oxygen present at a partial pressure of 10^−3^ atm (Ackermann et al., 2014a), and a δ as high as 0.3 with only 10^−7^ atm (Bulfin et al., 2013). In a further TGA study of CeO_2−δ_ for δ = 0.02–0.25, when ceria was reduced with an O_2_ partial pressure of 10^−4^ atm at 1,500°C and oxidized in CO_2_ between 400 and 1,000°C, it was found that a δ value of 0.034 was achieved after only 10 min, increasing to 0.057 after 180 min. To achieve greater levels of reduction, while avoiding sintering, the samples had to be heated to 1,100°C under a hydrogen partial pressure of 0.02 atm, arriving at a δ of 0.25 after 120 min. Following low thermal reduction of δ <0.06, oxidation rates under 0.1–0.2 atm of CO_2_ (and resultant CO production) slowly and linearly increased with the degree of initial non-stoichiometry (up to δ = 0.05), attributed to the formation of stable defect complexes between oxygen vacancies. With greater levels of reduction of δ = 0.06–0.2, achieved under H_2_, no significant change in oxidation rate was observed with changes in the extent of reduction, but the CO production rate was approximately an order of magnitude greater than that observed for the thermally reduced δ = 0.057 (Ackermann et al., 2015; Figure 5A). This was attributed to the creation of micro-cracks in the reduced ceria following chemical reduction under H_2_, which were absent in the thermally reduced ceria, and would effectively increase the available surface area. However, for δ > 0.2, oxidation rates dropped off considerably and rapidly (to values similar to those seen for δ = ~ 0.02), hindered by near-order changes such as lattice compression, which was confirmed with Raman Spectroscopy (Figure 5B). Importantly, this behavior was reversible and oxidation rates were not affected at lower δ.

Pure ceria was made by sol-gel, calcined at 800°C, and then tested in three cycles of reduction at 1,500°C for 30 min and oxidation with CO_2_ at 1,000°C for 45 min. However, this was performed in an electric tube furnace with long heating/cooling times of 10°C/min, resulting in a long total cycle period of ~175 min (Zhu et al., 2018), all at atmospheric pressure. This exhibited high and stable O_2_ and CO production over the three cycles, with the reduction step being slow and also occurring during the heating/cooling periods above 1,200°C, while the oxidation step was fast, and occurred only in the isothermal step where CO_2_ was injected into the furnace. This effectively means that the reduction time was actually 75 min between 1,200 and 1,500°C. This slow, enhanced reduction may be responsible for the high reported yields of 173–189 μmol_O2_ g^−1^ (~3.88–4.23 ml g^−1^) and 224–229 μmol_CO_ g^−1^ (~5.02–5.13 ml g^−1^), and shows how heating rates during the reduction step also contribute to the extent of the reduction process.

## Manipulation of Microstructure for Ceria Materials

**Figure 6:**
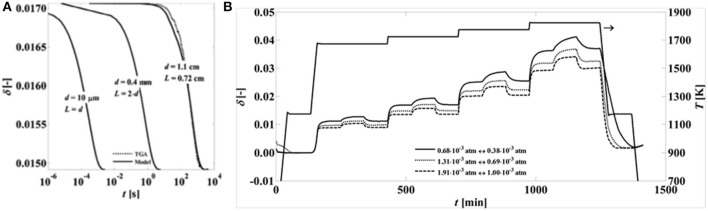
Effects of microstructure and temperature on non-stoichiometry of ceria. **(A)** Predicted reduction times for pure CeO_2−δ_ with varying diffusion length scales. The oxygen non-stoichiometries (δ) are predicted at 1,450°C with a pO_2_ swing from 0.69 × 10^−3^ atm to 1.31 × 10^−3^ atm. The samples have three different diffusion length scales: a cylinder (d = 1.1 cm, L = 0.72 cm), a strut of a reticulated foam (d = 0.4 mm, L = 2d), and a particle (d = 10 μm, L = d). **(B)** Variation of non-stoichiometry with temperature during reduction and oxidation, recorded over time for various oxygen partial pressure ranges. Adapted with permission from Ackermann et al. (2014a). Copyright 2014 American Chemical Society. Further permissions related to the material excerpted should be directed to the ACS.

It was shown that CeO_2_ reduction operates within a surface-controlled regime, as even for dense samples with a thickness of 1 cm oxygen diffusion times were in the order of hundreds of seconds, while at smaller diffusion length scales in the micrometer and millimeter range diffusion times were in the order of milliseconds to seconds, respectively (Ackermann et al., 2014a; see Figure 6). Other workers also reported surface dominant effects in the reduction of 93% dense sintered ceria of 1 mm thickness with 20 μm grains (Knoblauch et al., 2015). This was backed up by results from Chueh et al. (2010) and Furler et al. (2012b, 2014), who showed that the reaction rate-limiting factor was heat transfer during thermal reduction when using porous structures, due to their rapid reaction kinetics. Additionally, when micron-sized ceria particles were thermally reduced in an aerosol reactor, <1 s was required (Scheffe et al., 2014), again demonstrating that heat transfer, and not reaction kinetics, was the rate-limiting factor. It has also been seen that although ceria undergoes sintering and a loss of surface area at the high reduction temperatures, it can also convert from being microporous (pores <2–5 nm) to mesoporous (with pores around 20 nm) after redox cycles, suggesting that redox treatments could be employed to stabilize both pore structure and surface area against further sintering (Fornasiero et al., 1996). In pure CeO_2_, the occurrence of the redox process in the bulk induces formation of mesoporosity, which prevents further densification.

A study was made comparing the isothermal splitting of CO_2_ at 580°C in an electrically heated reactor by ceria nanoparticles (NPs), nanorods (NRs) and nanocubes (NCs) (Kovacevic et al., 2016). The NRs were 10 × 160 nm, while the NCs were 37 nm and the NPs were 26 nm, and the three had specific surface areas (SSAs) of 73, 37, and 24 m^2^ g^−1^, respectively. When mass specific CO production was considered, the NRs and NCs were very similar with rates of ~60–80 μmol min^−1^ g^−1^, the rate of the NRs dropping more over time than the NCs. The lattice expansion caused by increasing the amount of oxygen vacancies in the ceria fluorite lattice is known to create lattice microstrain, and theoretical studies predicted a decrease in the energy of oxygen vacancy formation with increasing lattice microstrain. From XRD data, lattice microstrain was much greater in the NCs (0.15%) than the NRs or NPs (both 0.08%), but the NRs had the larger SSA and showed a greater extent of oxygen vacancies in Raman measurements. Both of these features could explain the superior behavior of the NRs and NCs. It was also suggested that the superior CO production of the NCs could be due to the superior inherent reactivity of (100) crystal planes enclosing the NCs cubes, as opposed to the less inherently reactive (111) facets enclosing the NRs and NPs, and that the larger microstrain could be as a result of this, rather than itself causing the increase in CO production rates (Kovacevic et al., 2016).

Several porous ceria structures with morphologies on the μm-scale (such as macroporous materials, 3-DOM and felts) and mm-scale (such as honeycomb monoliths and foams) have been investigated (see sections 5–7 of this paper). Microporous structures such as monoliths or felts, with pore sizes in the μm range, have rapid oxidation rates thanks to their high SSAs, but are limited by their heat transfer rates as they absorb the incident heat radiation. This can cause large temperature gradients within the structure. However, macroporous structures with pore sizes in the millimeter, or even micron, range, such as foams and honeycombs, can experience uniform heating due to deeper penetration and bulk volume absorption of the concentrated solar radiation.

## Fibrous Ceria Materials

An early report of a fibrous ceria material used for solar thermochemical redox cycles was proposed: stable syngas production with tuneable H_2_:CO molar ratios was demonstrated using a commercial porous CeO_2_ felt insulation material (CeF-100 by Zircar Zirconia Inc.) in 2012 (Furler et al., 2012a), cycling between about 1,550 and 750°C with energy from a high flux solar simulator. This was for the combined production of CO and H_2_ (syngas) from CO_2_ and water, so the results cannot be directly compared to CO_2_ splitting alone, but with a high reduction temperature of 1,650°C for ~30 min and oxidation at ~1,000°C for 20 min, they produced 2.89 ml g^−1^ of O_2_ (corresponding to δ = 0.044) at a peak and average rate of 0.21 and 0.02 ml min^−1^ g^−1^, and 2.19 ml g^−1^of CO at a peak and average rate of ~0.6 and ~0.11 ml min^−1^ g^−1^. Over 10 cycles they produced 2.13–1.25 ml g^−1^ of O_2_ and 1.25–0.79 ml g^−1^ of CO, as the oxidation temperature was decreased from 1,580 to 1,495°C. The average solar-to-fuel energy conversion efficiency of 0.15% was considerably lower than the 0.4% average efficiency reported by Chueh et al. (2010), however, and this was attributed to heat transfer limitations from the low thermal conductivity of the CeO_2_ felt leading to inhomogeneous temperature distributions within the furnace.

The same commercial ceria fibers, were reported in another paper by the same group, using a high flux simulated solar light powered TGA, but at reduction and oxidation temperatures of ~1,325°C and ~825°C for short periods (5 min reduction, 4 min oxidation; Takacs et al., 2017). In this paper, the fibers were stated to be 7 μm diameter, 100 μm long, have 88% porosity and a density of 0.86 g cm^−3^. The fibers used for TGA were in the form of a disc 30 mm diameter and 24 mm high, so the volume was ~17 cm^3^, and its weight was 9.65 g, meaning the density (0.57 g cm^−3^) of the fibers shaped for solar TGA was about 2/3 that of the as-supplied fiber. They achieved a specific volume weight loss of ~2.3 mg cm^−3^ after 5 min. From this, it can be calculated that the mass specific weight loss was 1.31 mg g^−1^ (i.e., 0.13%). Afterwards, grain growth was seen over the entire surface of the sample, becoming more apparent where the ray of concentrated light was focused in the center of the top irradiated surface. On this spot, the fiber structure was completely destroyed with octahedral crystal grains as large as 30 μm forming.

In another study of commercial fibrous particles, of unstated origin, they evaluated over 1,000 isothermal CO_2_ splitting cycles (56 h) at 1,500°C (using only N_2_ as the reducing gas), followed by sixteen two-step temperature-swing cycles (5.7 h) with reduction at 1,500°C for 5 min and oxidation at 800°C for 4 min (Gladen and Davidson, 2016). The fibrous particles were 78% porous, with SSA = 0.143 m^2^ g^−1^ and a grain size of 3.7 μm, although these “fibers” were very short, with a diameter and length of 6.2 and 47 μm, respectively. With an aspect ratio of ~8 they were really whiskers, rather than fibers, which should have an aspect ratio >100.

During isothermal cycling, over the first cycle (3.33 min), 23% of the surface area was lost but changes in porosity and grain size were not measurable. Over cycles 2–25 (1.39 h) the average grain size increased to 5 μm, but additional surface area was not lost. This grain growth reinitiated surface area loss during cycle 26, and there was a consistent trend of decreasing SSA until it stabilized at 0.08 m^2^ g^−1^ during the 300th cycle. Grain growth stabilized after 500 cycles (28 h) at 8 μm, resulting in 73% porosity. There was little change in oxidation between cycles, with a very small cyclic change in non-stoichiometry δ of 0.0028, and an average CO production rate of only 0.9 μl s^−−s^ g^−1^ (= 54 μl min^−1^ g^−1^).

A low average CO production of 2.04 μl s^−1^ g^−1^ (= 0.120 ml min^−1^ g^−1^) was achieved over the first 50 cycles (2.78 h). CO production then decreased from the 50th cycle to the 300th cycle (16.67 h), to 1.82 μl s^−1^ g^−1^ (= 0.109 ml min^−1^ g^−1^), this 11% drop in CO production corresponding to a 27% loss in SSA from 0.11 to 0.08 m^2^ g^−1^. Over the next 700 cycles, fuel production and SSA remained stable, as were the peak CO production rates of 5 μl s^−1^ g^−1^ (= 0.30 ml min^−1^ g^−1^) at the onset of oxidation, and 3 μl s^−1^ g^−1^ (= 0.18 ml min^−1^ g^−1^) of CO after 100 s (Gladen and Davidson, 2016). This high retention of fuel production, despite a loss of SSA, was consistent with observations by others that fuel production is limited by thermodynamics rather than kinetics (Bader et al., 2013).

After one temperature-swing cycle (4 min) grain growth was evident (from 3.7 to 5.1 μm), and the SSA decreased from 0.143 to 0.093 m^2^ g^−1^, while the porosity (77%) did not change. To reach a similar SSA required 5.56 h of isothermal cycling, indicating that the temperature change, and the greater degree of reduction obtained (δ = 0.04 for the temperature swing cycle), has more effect than the maximum temperature attained, which was the same for both kinds of cycle. However, the peak CO production rate was two orders of magnitude higher, at 680 μl s^−1^ g^−1^ (= 40.8 ml min^−1^ g^−1^), and the average cycle production rate of 4.8 μl s^−1^ g^−1^ (= 0.288 ml min^−1^ g^−1^) of CO was also nearly three times that of the isothermal cycle. The average O_2_ production rate was ~2.5 μl s^−1^ g^−1^ (= 0.150 ml min^−1^ g^−1^), also nearly three times higher than the ~0.9 μl s^−1^ g^−1^ average achieved by the isothermal cycle. After 16 temperature-swing cycles, the SSA was further reduced to 0.057 m^2^ g^−1^, with a grain size of 8.7 μm and porosity of 72% (Gladen and Davidson, 2016). The larger swing in non-stoichiometry of temperature swing cycling can speed up sintering by decreasing the amount of oxygen vacancies (Djurovic et al., 2007) and by more rapid oxygen diffusion in the lattice (Inaba et al., 1998), and gradients in non-stoichiometry and temperature can also create strain due to differential expansion occurring within the particles. One other issue is that the authors also stated that the cost of these fibrous particles was high, at ~$6,000 per kg for a small production of 3 kg in 2016 (Gladen and Davidson, 2016) The manufacturing process for this material was similar to that used to produce high temperature refractory insulation, and based on discussions with the supplier, they would expect an order of magnitude cost reduction if the market for ceria fiber structures expands.

**Figure 7:**
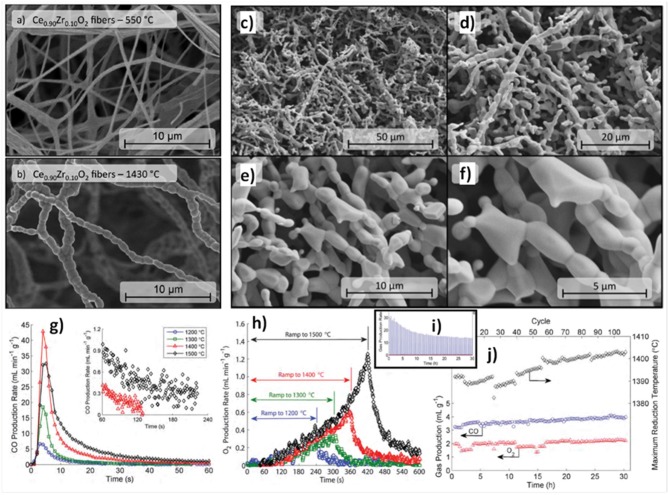
Thermochemical CO_2_ splitting with 0–10 mol% Zr doped electrospun ceria fibers. Ce_0.9_Zr_0.1_O_2_ fibers **(a)** after heating to 550°C in air and **(b)** after heating to 1,430°C in air for 6 h. SEM images [1, 2, 5, and 10 k magnification for images **(c–f)**] of Ce_0.975_Zr_0.025_O_2_ fibers following the long-term stability test with T_red_ = 1,400°C for 108 cycles. **(g)** CO production rates vs. time for the 10th cycle for Ce_0.975_Zr_0.025_O_2_ fibers for different T_red_. Inset plot shows the final 3 min of the CO production step for the samples reduced with T_red_ = 1,400°C and 1,500°C, indicating that the sample reduced at 1,500°C may not return to equilibrium in the time provided for oxidation. **(h)** O_2_ production rate vs. time for the 10th cycle for Ce_0.975_Zr_0.025_O_2_ fibers for different T_red_. The plot shows O_2_ production during both the temperature ramp to T_red_ and the hold at T_red_. Time is zeroed at the beginning of the ramp from 800°C up to T_red_. **(i)** Trend in CO production rate for Ce_0.975_Zr_0.025_O_2_ fibers during 108 complete redox cycles with T_red_ = 1,400°C, showing a sharp peak at the beginning of the run which decays asymptotically with increasing cycle number due to fiber sintering and loss of surface area. **(j)** Time-integrated total CO and O_2_ production (in ml g^−1^) and actual maximum T_red_ for Ce_0.975_Zr_0.025_O_2_ fibers over 108 cycles with Tred = 1,400°C. Reproduced from Gibbons et al. (2014) with permission from the PCCP Owner Societies.

A study was carried out on 0–10 mol% Zr doped electrospun ceria fibers, heated for 10 cycles in an infrared furnace with reduction at up to 1,500°C/5 min and oxidation at 800°C/4 min, after an initial reduction stabilization step of 1 h (Gibbons et al., 2014). An example of the fibers, as made at 550°C and after heating to 1,430°C/6 h is shown in Figures 7a,b, and it can be seen that the grains remained well below 1 μm. Ce_1−x_Zr_x_O_2_ fibers with x = 0.0, 0.025, 0.05, and 0.10 were put through 10 thermochemical cycles, with reduction at 1,200°C for x = 0 and 1,140–1,160°C for x = 0.025–0.10, and oxidation at 800°C and 740–760°C. The quantity of CO produced increased from 0.40 ml g^−1^ for the pure ceria to between 0.98 and 1.60 ml g^−1^ as x increased from 0.025 to 0.10, and δ showed a similar increase, from 0.0028 for pure ceria, to 0.0069–0.0120 with x = 0.025–0.10.

The incorporation of ZrO_2_ into the CeO_2_ framework with formation of a solid solution (e.g., Ce_0.5_Zr_0.5_O_2_) does strongly affect the redox behavior, since the reduction occurs concurrently at the surface and in the bulk of the solid solution (Fornasiero et al., 1996). However, as shown with Zr doped ceria ceramics (Furler et al., 2012b, 2014) although yield increased with doping, the oxidation rate greatly decreased, with a slower initial production and longer period needed for completion. Thence, the 2.5 mol% Zr composition (Ce_0.975_Zr_0.025_O_2_) was selected for further thermochemical studies with short cycles because it achieved similar rapid CO production rates to undoped ceria, but underwent a greater degree of reduction, and retained a smaller crystallite size, signifying improved sintering resistance. This can be seen in Figures 7c–f, which shows the x = 0.025 fibers following a long-term stability test with reduction at 1,400°C for 108 cycles.

When put through 10 cycles with reduction steps between 1,200 and 1,500°C, the CO yield was stable within ±10%. The CO and O_2_ production for the 10th cycle are shown in Figures 7g,h. The peak CO production rate at 800°C increased from 7 to 17 and then 43 ml min^−1^ g^−1^ as the reduction temperature increased from 1,200 to 1,400°C, but fell slightly at 1,500°C to 32 ml min^−1^ g^−1^. However, the total CO yields rose monotonically with reduction temperature, being 0.88, 1.6, 4.7, and 7.0 ml g^−1^ for reduction at 1,200, 1,300, 1,400, and 1,500°C. This fall in CO production rates was observed despite the fact that total CO yield, O_2_ production and degree of reduction continued to increase with increasing reduction temperature (Figure 7h). This was explained by considering the fraction of exposed surface vacancies, which increased from a δ of 0.006 to 0.049 as reduction temperatures rose, and the fiber SSA, which was found to drop steadily from 3.17 to 0.28 m^2^ g^−1^ from 1,200 to 1,500°C, decreasing by slightly more than a factor of 2 for each 100°C rise in reduction temperature. Thus, the peak CO production rate was lower with reduction at 1,500°C, even though the total CO yield was higher, because the increase in vacancy concentrations due to increased δ failed to continue to outweigh the loss of SSA at 1,500°C (Gibbons et al., 2014). As reduction at 1,400°C gave the greatest CO production rates, this was chosen for measurements of 108 cycles, which had an average δ of 0.027 and an average CO yield of 3.9 ml g^−1^, ~82% of the single cycle values at that temperature. It can be seen in Figure 7i that the CO production rate fell with increasing cycles as SSA continues to shrink, but that total CO yield actually continually increased (~20% for all 108 cycles) at the same time (along with O_2_ yield; Figure 7j). This was because the measured maximum reduction temperature actually also rose slightly with cycles from 1,390 to 1,405°C, indicating that with further sintering they became more efficient at absorbing/transferring the heat into the redox process. These results emphasize the complex interplay of δ, porosity, grain size and SSA involved in determining the capability and efficiency of ceria materials in splitting CO_2_.

## Three-Dimensionally Ordered Macroporous (3-DOM) Ceria

**Figure 8:**
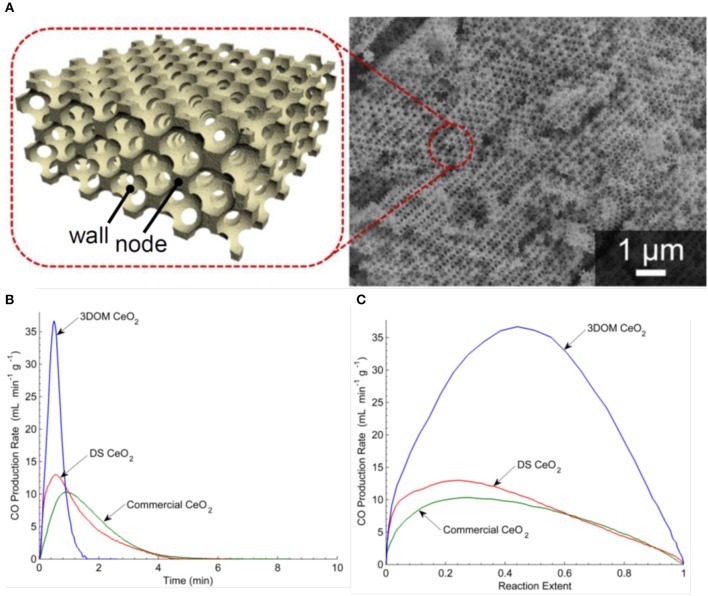
Three-dimensionally ordered macroporous (3-DOM) honeycomb-like ceria ceramics produced from a template of PMMA spheres. **(A)** Computer rendering and SEM images 3-DOM CeO_2_. For an isothermal cycle at 800°C, CO production rates as a function of **(B)** time and **(C)** reaction extent for CO_2_ splitting for commercial ceria, DS ceria and 3-DOM pure ceria. Adapted from Venstrom (2012), ©Luke J. Venstrom.

In 2011, three-dimensionally ordered macroporous (3-DOM) honeycomb-like ceramics were produced, made of CeO_2_ and Ce_0.8_Zr_0.2_O_2_ (Venstrom et al., 2011). Colloidal crystal templates with mm size dimensions were made from 415 nm poly(methyl methacrylate) (PMMA) spheres, which naturally self-assembled into a face-centered cubic (fcc) close-packed array upon sedimentation. These were infiltrated with precursor solutions, and the dried polymer sphere template and solidified precursor composites were heated to 450°C to remove the polymer spheres, leaving an inverse replica 3-DOM structure (sometimes known as inverse opals) formed of nanocrystalline pure or doped ceria. These were then broken into pieces, sieved to sizes <1 mm, and mounted in a fixed bed reactor in an electric furnace for thermocycling tests. Two other ceria materials lacking the fcc pore network of the 3-DOM ceria were also compared to it—a random, foam-like ceria from decomposition synthesis (DS), and large 3–6 mm commercial ceria pieces. Before thermochemical cycling, the large ceria pieces had a relatively low SSA of 1 m^2^ g^−1^, the DS ceria had a very high SSA of 112 m^2^ g^−1^, and the ceria and Zr doped ceria 3-DOM materials were 30 and 25 m^2^ g^−1^, respectively. A 3D render and a SEM image of the 3-DOM ceria are shown in Figure 8A. An isothermal cycle was used at a constant temperature of 800°C and using a mixture of 5% H_2_ in Ar to reduce the samples for ~30 min followed by the addition of 4% CO_2_ in Ar for about 1 h. As this is an isothermal cycles with forced reduction, it is difficult to compare it with the more standard two step cycles operated at two different temperatures. After reaction, the SSA of the large ceria pieces had not changed, the DS ceria lost 99% of its SSA during reduction to a much lower 1.1 m^2^ g^−1^, and the pure and doped 3-DOM ceria had lower, but still reasonable, SAAs of 10 and 31 m^2^ g^−1^, respectively.

The O_2_ production rates are not given (because of the H_2_ induced reduction), but the CO production is shown in Figures 8B,C. Not surprisingly, as they had very similar SSA values for the oxidation step, the commercial ceria pieces and DS ceria had very similar profiles, with peak CO production rates of 10.4–13.6 ml min^−1^ g^−1^, an average production rate of 6.8–7.7 ml min^−1^ g^−1^, and a total CO yield of 23 ml g^−1^. Surprisingly, despite the much higher surface areas, the 3-DOM cerias had slightly lower total CO yields of 22 ml g^−1^, but much quicker peak CO production rates of 37.4–51.2 ml min^−1^ g^−1^ and average production rates of ~22 ml min^−1^ g^−1^. The induced reduction with H_2_ resulted in a δ of 0.16 for CeO_2−δ_, an order of magnitude higher than that achieved with thermal reduction alone, so the absolute quantities produced cannot be compared to standard two step solar splitting. However, the 3-DOM structure had an effect on CO fuel production rate, increasing it by a factor of ~5 with pure ceria, and ~6.5 for Ce_0.8_Zr_0.2_O_2_ (Venstrom et al., 2011). It was found that for pure ceria, the interconnected and ordered pores increase the maximum CO production rate over low porosity ceria by 260%, and increased the maximum CO production rate over non-ordered mesoporous cerium oxide by 175%. The enhanced SSA of the 3-DOM ceria and its interconnected pore system, which facilitated the transport of reacting species to and from oxidation sites, was given as the cause of this increase in the kinetics of CO_2_ splitting (Venstrom et al., 2012). This was the first work to clearly demonstrate the benefits of a regular 3-DOM network structure on CO production rates, albeit with quite small cells of only ~0.4 μm (Venstrom, 2012).

**Figure 9:**
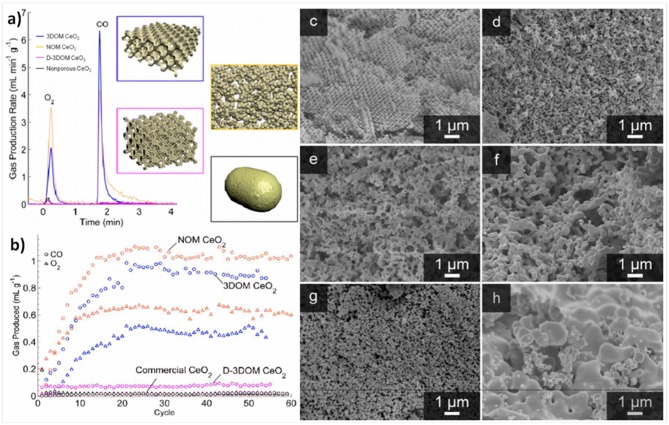
3-DOM, non-ordered macroporous (NOM), and aggregated particles of the 3-DOM material (D-3DOM) ceria, and their use in thermochemical CO_2_ splitting. **(a)** 3D renders of the 3-DOM, random NOM, D-3DOM nanoparticle and commercial cerias used, and a typical stabilized thermochemical cycle with O_2_ and CO_2_ production rates (reduction at 1,200°C, oxidation at 850°C. **(b)** Total O_2_ (triangles) and CO (circles) produced in each thermochemical cycle over 3-DOM, NOM, D-3DOM, and commercial CeO_2_. Each cycle only lasted 3 min, with very short reduction and oxidation steps of 78 and 90 s each. SEM images of CeO_2_ materials before (left) and after (right) cycling: For 3-DOM before **(c)** and after cycling **(d)**, although the walls have undergone significant sintering and in some areas the order has been lost, the pore structure remains interconnected. For NOM before **(e)** and after cycling **(f)**, the most prominent change after cycling is an increase in skeletal wall thickness—otherwise, the overall morphology of the structure remains unchanged. For D-3DOM before **(g)** and after cycling **(h)**, it is clear that the sample has undergone extensive sintering. Reprinted with permission from Rudisill et al. (2013). Copyright 2013 American Chemical Society.

In a recent paper, the same authors further investigated the effects of porosity, pore order and packing density of 3-DOM ceria made with 515 nm PMMA spheres. This inverse opal network consisted of 500 nm voids contained within 92 and 189 nm nodes (from interstitial tetrahedral and octahedral sites in the fcc template), connected by 48 nm struts. The 3-DOM ceria was compared to commercial ceria pellets, non-ordered macroporous (NOM) CeO_2_, and aggregated particles of the 3-DOM material (D-3DOM) prepared by fragmenting it into nano- to 3 μm sized particles using ultrasonication (Rudisill et al., 2013). The average wall thickness separating the voids in NOM was 172 ± 118 nm, and the D-3DOM consisted of 90% anisotropic nanoparticles (a “dust” made from the remains of the tetrapodal and octapodal nodes) clustering around and coating particles of intact 3DOM structure with a maximum particle size of ~3 μm. 3D renders of these morphologies are shown in Figure 9a. These were all tested in an infrared furnace, using thermochemical cycles to split CO_2_ with reduction at ~1,200°C and oxidation at ~850°C, for 60 cycles. Due to the very quick heating rate of the IR furnace, and the rapid reaction rates of the porous materials tested, each cycle only lasted 3 min, with very short reduction and oxidation steps of 78 s and 90 s each. Before cycling, the 3-DOM, NOM, D-3DOM, and commercial cerias had surface areas of 47.6, 47.3, 95.7, and <1 m^2^ g^−1^, and total pore volumes of 0.412, 0.111, 0.318, and 0.002 cm^3^ g^−1^, respectively. However, after cycling, the values had all fallen considerably for the porous materials, while they did not change for the commercial ceria. After 60 cycles, all of the porous cerias had SSA of ~4 m^2^ g^−1^, and pore volumes of ~0.01 cm^3^ g^−1^. SEM images of the samples before and after cycling are shown in Figures 9c–h.

The rate of production for an average redox cycle after stabilization for the various ceria morphologies is shown in Figure 9a, and the total O_2_ and CO production of each cycle is shown in Figure 9b. The increase in production over the first 20 cycles is because the ceria required this number to become fully reduced at only ~1,200°C (a total reduction time of 26 min). Despite their virtually identical SSAs, the macroporous 3-DOM and NOM ceria behaved very differently to the D-3DOM, which was almost as poor as the commercial ceria in terms of O_2_ and CO production. The two macroporous forms immediately exhibited higher production yields per cycle, which then increased steadily for the first ~20 cycles, until they were producing an order of magnitude more than the commercial and D-3DOM cerias. After around 30 cycles (full stabilization), CO production yields were 0.9, 1.0, 0.08 and 0.06 ml g^−1^ for 3-DOM, NOM, D-3DOM, and commercial ceria, respectively. These values for 3-DOM and NOM of ~1 ml g^−1^ for a whole cycle which only took 3 min are impressive, and are due to the very high production rates attained with the macroporous cerias. Despite having a slightly lower yield, the rate of CO production was higher for 3-DOM (0.83 ml min^−1^ g^−1^) than for NOM (0.50 ml min^−1^ g^−1^), and it was only 0.03 ml min^−1^ g^−1^ for the other two cerias. It should be noted that these rates were also obtained with a reduction temperature of no more than 1,200°C, but they compare to other porous cerias with reduction at 1,400–1,500°C. As 3-DOM and NOM ceria had similar yields, with NOM actually being slightly superior, this demonstrated that the degree of macropore periodicity did not influence CO production. However, the more periodic 3-DOM structure led to greater interconnected porosity, improving the kinetics of the oxidation step, with faster CO evolution. Furthermore, the 3-DOM structure had a greater resistance to sintering than other morphologies, as the overall contact between grains was reduced by the macropores (Rudisill et al., 2013).

Other modeling studies on 1 μm 3:DOM ceria particles with smaller 330 nm interconnected pores in a FCC arrangement were carried out (Wheeler et al., 2014). Compared to bulk ceria, such particles canceled out wave extinction for wavelengths >560 nm (still within the visible light spectrum, and into the near-IR), and the wavelengths were required to be much greater than the pore size. Particle orientation and non-spherical shape also have a large effect on their radiative properties. Vis/near-IR measurements (350–2,200 nm) were carried out on powdered 3-DOM ceria with 485 nm pores and interconnecting windows of 14 nm, packed in beds of varying thickness (0.57–1.18 mm), which were subjected to thermochemical cycling between 1,100°C/1 min and 800°C/2 min between measurements. Porosity before thermocycling was 0.74, and after it had actually increased to 0.83. This was also compared to sintered bulk ceria (porosity = 0.08) and another sintered porous ceria (porosity = 0.72). With the 3-DOM ceria, it was found that transmittance decreased significantly with increasing thickness over whole range measured, while reflectance and absorbance showed much smaller variations. All 3-DOM samples exhibited similar peaks in IR absorbance at ~1,400 and ~2,000 nm, and similar scattering coefficients between 10 and 20 nm^−1^ in the IR range of 700–2,200 nm (and a large degree of scattering in the visible region). Absorbance increased, but reflection, and scattering coefficient decreased only slightly after the 3-DOM ceria was thermocycled, while there was a significant increase in the transport scattering coefficient, and it was superior to the other sintered porous ceria (Ganesan et al., 2013). This suggests that if the 3-DOM structure can be preserved during thermochemical cycling, it will lead to scattering characteristics that can allow longer attenuation path lengths of incident concentrated solar radiation within the material. This should favor the confinement of near-IR radiation during thermochemical cycling, aiding thermochemical fuel production.

## Reticulated Ceria Foams

An alternative way to a highly porous ceria structure is to use a CeO_2_-based reticulated porous ceramic (RPC) foam, with a net-like structure. This is not an ordered microstructure, so it is not a 3-DOM material. These may have dual-scale porosity, with mm-sized pores offering better radiative heat transfer during reduction, and micron-sized pores within the struts giving enhanced oxidation kinetics. They are most easily made by coating a pre-existing metal or ceramic structure with the catalytic or redox phase, but this can undergo side reactions between the redox material and the support during sintering and thermocycling, with spallation and/or deactivation as a consequence (Furler et al., 2012b).

**Figure 10:**
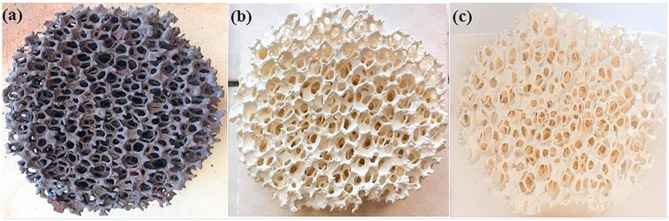
Ceria reticulated porous ceramic (RPC) foam **(a)** after ceria precursor/powder coating (15 mm thickness, 63 mm diameter), **(b)** after firing at 1,000°C/6 h (14 mm thickness, 60 mm diameter) and **(c)** after final heat treatment at 1,050°C/5 h and sintering (13 mm thickness, 55 mm diameter). From Chuayboon et al. (2019), used with permission from Elsevier.

A superior method is the replication technique, using a polymer foam template which is coated with a ceria precursor. Often a pore forming agent such as carbon powder is also added, to confer the micron scale porosity within the struts and bulk of the RPC foam. The treated foam is then heated to a high temperature to burn out the polymer template, leaving behind a replica ceramic structure, such as those depicted in Figures 10a–c, used in both the production of syngas and the splitting of CO_2_ and H_2_O (Chuayboon et al., 2019).

**Figure 11:**
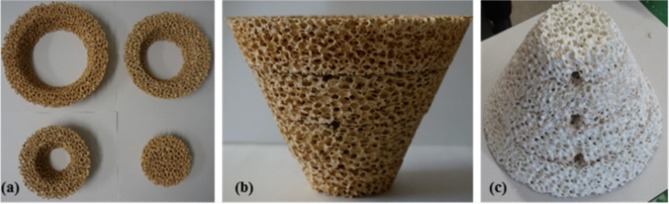
Photographs of a conical RPC foam thermochemical reactive material, made from a four-section Mg-stabilized zirconia structure **(a,b)**, which was then coated with 40 wt% ceria powder, which is a white color **(c)**. The three holes are for the insertion of thermocouples. From Cho et al. (2017), used with permission of AIP publishing.

RPC foams can be made in other, more complex shapes as well, such as the conical piece (a magnesium stabilized zirconia foam coated with 40 wt% ceria) made from 4 sections shown in Figure 11, which was successfully used to split water using actual concentrated sunlight (Cho et al., 2017).

**Figure 12:**
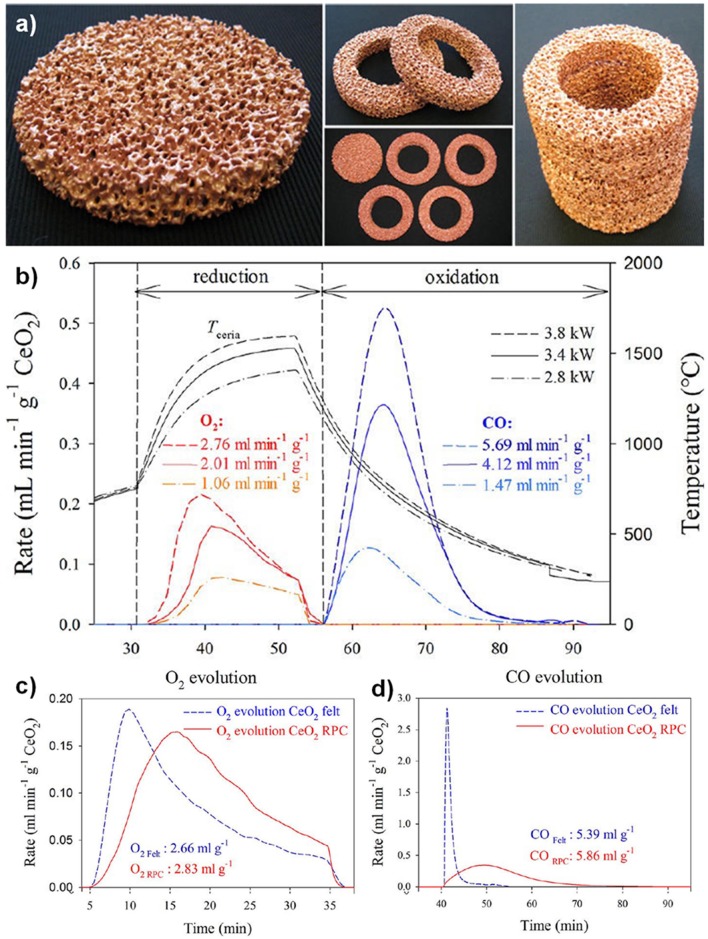
Thermochemical CO_2_ splitting with a reticulated porous ceramic (RPC) ceria on a High-Flux Solar Simulator **(a)** CeO_2_ RPC foam parts fabricated for the solar cavity-receiver. One set consists of a disk and four rings, combining to form a hollow 100 × 100 mm cylinder. **(b)** Nominal reactor temperature and O_2_ and CO evolution rates during three individual redox cycles for different solar radiative power inputs during the reduction step. Production rates, and total yield, of **(c)** O_2_ and **(d)** CO from RPC ceria foams, compared to ceria felt (mass = 1,413 g for RPC, 90 g for felt). Adapted with permission from Furler et al. (2012b). Copyright 2012 American Chemical Society.

The first reports of a ceria RPC foam for solar thermochemical CO_2_ splitting were in 2012, by the groups from ETH Zurich, the EMPA Laboratory for Hydrogen and Energy, and the Solar Technology Laboratory of the Paul Scherrer Institute, all based in Switzerland (Furler et al., 2012b). They made a ceria RPC foam by the replication method, from commercial polyurethane sponges, and sintered at 1,600°C. The foam had a low SSA <0.1 m^2^ g^−1^ despite its large scale macro porosity (estimated to be 1.45 × 10^−4^ m^2^ g^−1^ from tomography), the struts between the macro pores being in the order of 1 mm thick. A photograph of the RPC ceria foams is shown in Figure 12a. This was tested in a solar reactor under high flux simulated solar light (between 2.8 and 3.8 kW), with a reduction step of 22 min, followed by an oxidation step of ~40 min. The solar energy used, temperatures, production rates, and total yields can be seen in Figure 12b. A non-stoichiometry of δ = 0.016, 0.031, and 0.042 for CeO_2−δ_ was achieved in the three tests, increasing with solar radiation/temperature. From these results, the maximum theoretical η (solar to fuel efficiency) was calculated to be 0.73, 1.44, and 1.73%, for reduction steps lasting 22 min at 2.8 kW (average temperature of 1,420°C), 3.4 kW (1,530°C), and 3.8 kW (1,600°C). This resulted in total CO yields of 1.465, 4.107, and 5.690 ml g^−1^, which are high values (Figures 12c,d). The enhanced heat transfer capabilities and greater bulk density of the RPC foam compared to ceria felt also resulted in a significant increase in the absolute rate of O_2_ evolution (as much more reactive ceria was contained in the same volume compared to felt), and lower temperature gradients throughout the structure, increasing the average efficiency by a factor of over 10, as the same volume of RPC contained 1,413 g of ceria, compared to just 90 g for the felt. However, the oxidation kinetics were limited by the relatively low SSA, with the oxidation reaction taking over four times longer to reach completion in the denser RPC than that of the felt, and reaching much lower peak CO generation rates.

**Figure 13:**
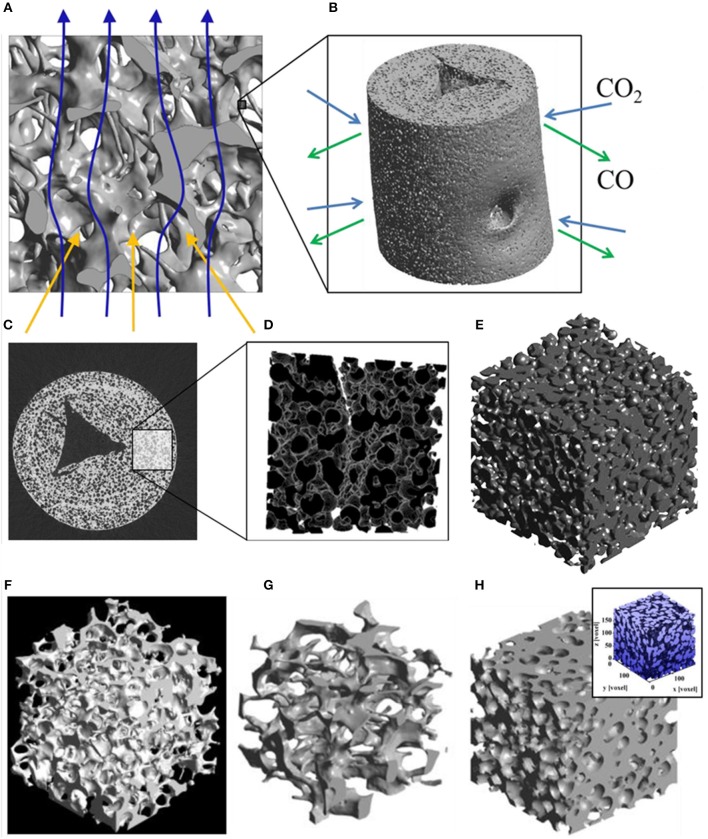
3D model of a ceria RPC foam with dual-scale porosity showing **(A)** mm-size pores for volumetric radiative absorption and effective heat transfer during the reduction step, and **(B)** connecting struts containing micron-sized pores leading to increased specific surface area for enhanced reaction kinetics during the oxidation step. **(C)** Synchrotron submicrometre computer tomogram of a cross section of a single RPC strut made with 50 vol% pore former concentrations, **(D)** a 3D rendering of the strut microstructure showing high porosity, and **(E)** the corresponding 3D digital reconstruction of the void space within an isotropic porous strut region. Digital renders showing the structure of **(F,G)** the RPC ceria foam with mm sized pores, and **(H)** the microstructure of the connecting struts with micron scale pores. From Ackermann et al. (2014b), used with permission.

To address this problem, these authors developed a series of CeO_2_-based RPC structures with dual-scale porosities in order to combine the improved heat transfer enabled by mm-sized pores and improved oxidation kinetics resulting from micron-sized pores within the supporting struts (Furler et al., 2014). A detailed 3D digital representation of the complex dual-scale porosity found in these RPCs is shown in Figure 13, obtained using synchrotron submicrometre tomography and X-ray microtomography measurements of the ceria RPC foam reported in Venstrom et al. (2012), made with between 10 and 50 vol% of carbon pore forming agent added to vary internal porosity. Total and open porosity, mean pore diameter, pore size distribution, and SSA were extracted from the CT scans, and this 3D digital geometry was then applied in direct pore level simulations for the accurate determination of the effective thermal conductivity at each porosity scale, with a wide range of porosities of 0.09–0.9%, including both the μm-scale in the struts and mm-scale in the bulk (Ackermann et al., 2014b).

Figures 13A,B depicts the effects of mm scale and micron scale porosity on the reduction and oxidation steps, respectively. Submicron tomography of the connecting struts showed that they contained a large closed void in the center, left over from the removal of the polymer template (Figure 13C). Apart from this, the connecting struts were seen to contain many pores, averaging 9 μm diameter (Figure 13D), and with 50 vol% pore former the struts had an open porosity of about 40%, giving a calculated SSA of 0.04 m^2^ g^−1^. With 30 vol% pore former there was still some closed micron scale porosity within the struts, but with 50% it was all totally open, as can be seen in the 3D render of open pores in Figure 13E, giving the whole RPC foam an open (micron & mm scale) porosity of around 80%. What can also be observed in Figure 13C is a ring within the cross section of the strut, a bit like that of a tree, and in the render in Figure 13D an actual crack-like formation can be seen. This is because the coating was applied to the polymer template in two layers, effectively creating a barrier between them, emphasizing the effect that variations in the synthesis and processing of such structures can have. Digital renders showing the hierarchical, dual scale nature of the porosity of the RPC ceria foam, with mm sized pores in the bulk, and micron sized pores in the connecting struts, are shown in Figures 13F–H (Ackermann et al., 2014b). Another recent modeling paper has suggested that for transient heat transfer through a ceria RPC foam, a porosity of 0.75 with a pore size of 2.2 mm showed a good compromise between high specific mass load and moderate optical thickness and permeability, showing the greatest oxygen yield per ceria mass. Also, a RPC structure with two macropore size regions, with large pores of 2.2 mm for the front part and small pores of 0.6 mm in the rear part, achieved the highest solar-to-fuel energy conversion efficiency, with the highest peak of η = 0.9%, due to reduced radiation losses at the rear (Ackermann et al., 2017).

**Figure 14:**
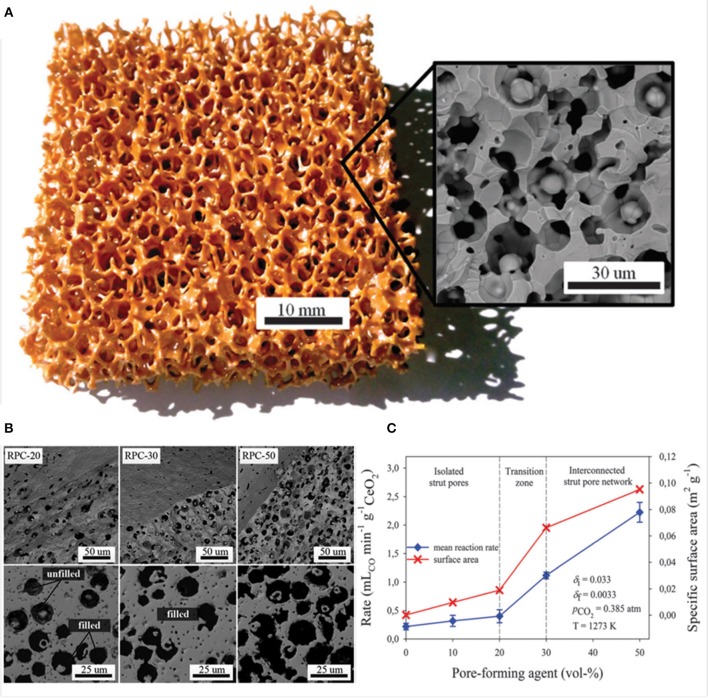
RPC ceria foams with dual-scale porosity and the effects of pore size/number on CO production. **(A)** Photograph of a RPC ceria foam with dual-scale porosity produced with 50 vol% of pore-forming agent. Inset: SEM micrograph of a break plane of its struts. **(B)** SEM micrographs of the surface and break plane (top), and back-scattered electron detector SEM images of polished cuts through a single strut (bottom), for of RPC ceria produced with 0–50 vol% pore-forming agent. **(C)** Mean CO evolution rate during the oxidation step and SSA as a function of pore-forming agent vol% for RPC samples following thermal reduction at 1,500°C. Reproduced from Furler et al. (2014) with permission from the PCCP Owner Societies.

A photograph and SEM image of one of these dual scale ceria RPC foams, made with 50 vol% pore former, is shown in Figure 14A. In a follow-up paper (Furler et al., 2014), ceria RPC foams were produced with between 0 and 50 mol% added carbon pore forming agent. SEM images of the foams with 20, 30, and 50 vol% pore former and sintered at 1,600°C are shown in Figure 14B, depicting the progression in microstructure with increasing quantities. Most of the pores remained close with up to 20 vol%, but almost all were open and interconnected with 30 and 50 vol%, and independent of quantity of carbon, all foams contained smaller 2 μm pores due to incomplete sintering or enclosure of generated gasses. The large scale open porosity of the bulk foams remained virtually constant at around 80% irrespective of carbon addition, but the microscopic open porosity of the struts increased greatly, from zero with no carbon added to 18 and 40% with 30 and 50 vol%, as can be observed in the back scattered SEM images in Figure 14B. The SSA also increased considerably, from 0.00015 m^2^ g^−1^ with no carbon added to 0.019, 0.066, and 0.095 m^2^ g^−1^ for the foams with 20, 30, and 50 vol%, respectively, as shown in the surface area data in Figure 14C.

**Figure 15:**
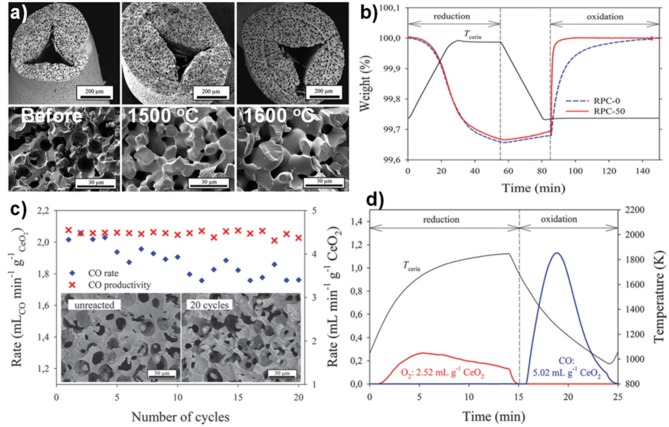
The internal microstructure of the connecting struts in RPC ceria foams, and its effects on CO production. **(a)** SEM micrographs of strut cross section for RPC-50 before, and after 120 h at 1,500 and 1,600°C in air. **(b)** TGA CO_2_ splitting redox cycle for RPC-0 (dashed line) and RPC-50 (solid line) with reduction at 1,500°C and oxidation at 1,000°C. **(c)** Mean CO production rates and CO yield per cycle for RPC-50, for 20 consecutive TGA redox cycles with reduction at 1,500°C and oxidation at 1,000°C, and SEM micrographs before (left) and after (right) the 20 cycles. **(d)** Redox cycle performed in a solar cavity-receiver exposed to high flux simulated solar radiation during the reduction step on RPC-30. Reproduced from Furler et al. (2014) with permission from the PCCP Owner Societies.

These foams were put through a TGA redox cycle, with reduction at 1,500°C for 30 min and oxidation at 1,000°C for 60 min (Furler et al., 2014). The mean rate of production of CO in the oxidation step is also shown in Figure 14C, following exactly the trend in SSA, and with a large jump in mean rate to around 2.0 and 2.5 ml min^−1^ g^−1^ as the micron scale pores became open with 30 and 50 vol% carbon added. To gauge the effects of long-term exposure to these cycling temperatures on the foams, that with 50 vol% pore former added (RPC-50) was heated in air at 1,500 and 1,600°C for 120 h (Figure 15a). The integrity of the strut was maintained and the interconnected network was not affected, although there was significant grain growth, with SSA decreasing from 0.095 m^2^ g^−1^ to 0.056 and 0.036 m^2^ g^−1^ after 120 h at 1,500 and 1,600°C, respectively. The redox cycles for RPC-50 and RPC-0 (no carbon added) are shown in Figure 15b, and it was observed that there was no difference between the two during the reduction step, proving that this was dependent on the large scale bulk porosity of the foam (enabling good thermal transfer), and not the microstructure of the struts. Both began to reduce at 1,000°C, and achieved an overall CeO_2−δ_ reduction value of δ = 0.037. There was a small weight gain for both during cooling in Ar to the oxidation temperature of 1,000°C due to partial re-oxidation with residual O_2_ in the system. However, upon introduction of CO_2_, the two foams behaved very differently, the initial oxidation rate for RPC-50 being very rapid, reaching 90% after 3 min, an order of magnitude greater than for RPC-0 (2.22 vs. 0.22 ml min^−1^ g^−1^), proving that the increase in SSA with the induced open micron-scale porosity in RPC-50 was key in enabling a rapid oxidation, although after 60 min both had become virtually fully re-oxidized (Furler et al., 2014).

The mean CO production rate (Figure 15c) obtained with RCP-50 (2.2 ml min^−1^ g^−1^) was much greater than that originally obtained by Chueh et al. (0.105 ml min^−1^ g^−1^) with porous monolithic ceria of SSA = 0.1 m^2^ g^−1^ under similar conditions (Chueh et al., 2010), and also than that obtained with the original ceria RPC foams with no pore forming agents added (Furler et al., 2012b), which had mean values of 0.064–0.140 ml min^−1^ g^−1^ depending on heat input, despite having a similar maximum δ of 0.035. It is to be expected that further increasing the SSA of the RPC foam would result in even higher rates than these, but at the expense of reducing the density for a fixed volume of ceria. As solar reactors currently contain a fixed volume of ceria (without the continuous feeding or removal of ceria), increasing SSA effectively decreases the total mass of reactive material available, lowering the possible fuel output per cycle. For this reason, optimization of porosity is only one of several factors that need to be taken into consideration to maximize the solar-to-fuel conversion efficiency.

These authors also tested RCP-30 under high flux simulated solar light of 3.8 kW (~3,000 suns) at a heating rate of up to nearly 200°C min^−1^, with reduction occurring between 1,000 and 1,575°C over 15 min, and oxidation between ~1,300 and 825°C over 10 min (Figure 15d) with no light applied (natural cooling). Peak and average O_2_ production rates of 0.27 and 0.17 ml min^−1^ g^−1^ were obtained, respectively, with a total O_2_ production of 2.52 ml g^−1^ (equal to δ = 0.039). For the oxidation step, a rapid peak CO production rate of 1.13 ± 0.17 ml min^−1^ g^−1^ and an average CO production rate of 0.63 ± 0.17 ml min^−1^ g^−1^ was achieved, for a total CO yield of 5.02 ml g^−1^, a CO:O_2_ ratio of 1.99, implying that δ was fully exploited for fuel production. A mean solar-to-fuel efficiency of 1.72% was achieved, equal to other results using standard RPC foam in the same reactor (same volume of ceria) but with 50% more mass of ceria (Ackermann et al., 2014b), showing that a higher solar efficiency could be attained with the dual scale porosity RPC foams (Furler et al., 2014).

This hierarchical dual porosity RPC CeO_2_ foam was also used to produce 700 L of syngas (from 291 cycles) in Switzerland, which was then compressed, stored, and transported to Shell Global Solutions in Amsterdam, where it was converted into the world's first ever solar-derived kerosene (albeit actually using high flux simulated solar light) via the Fischer-Tropsch catalytic process (Marxer et al., 2015; Roeb et al., 2016). In 2017, an even better solar-to-fuel energy conversion efficiency of 5.25% was achieved using a CeO_2_-based dual-scale porosity RPC foam, in a second generation solar cavity reactor (still using high flux simulated solar light) that improved the heat and mass transfer characteristics of the system (Marxer et al., 2017). Indeed, the overall kinetics of the redox cycle were found to be controlled by heat and mass transfer within the solar reactor, and not by solid-state diffusion within the crystal lattice of ceria.

**Figure 16:**
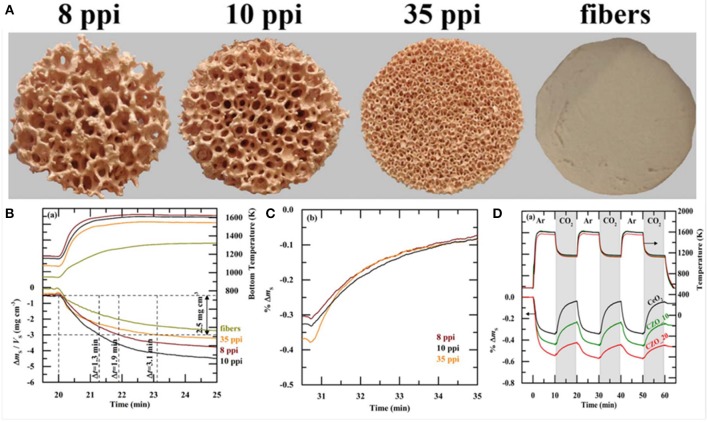
Photographs of ceria DS-RPC structures with 8, 10, and 35 ppi in the mm-scale and 18% porosity within the struts in the μm-scale **(A)**. Also shown is the ceria fiber sample with 88% porosity in the μm-scale. All are ~30 mm diameter, and the RPCs were ~18 mm thick. Solar TGA measurements of the 8, 10, and 35 ppi ceria DS-RPC and the ceria fiber sample: **(B)** temperature at the bottom of the sample and specific weight change as a function of time during reduction (~1,325°C; **(C)** % weight change during oxidation with CO_2_ at 875°C. **(D)** % mass change and temperature of CeO2, CZO-10 and CZO-20 DS-RPC with 10 ppi during the three redox cycles. From Takacs et al. (2017), used with permission.

At the same time, in 2017 RPC ceria foams and ceria fibers (fiber results discussed above) were compared in a high flux solar simulator driven TGA. Ceria doped with 0–20 mol% Zr was studied, and RPCs with various macro pore sizes, as well as RPCs with single scale (SS) and dual scale (DS) porosities (Takacs et al., 2017). The RPC foams were made as above, but three different polyurethane foams were used as templates, with 8, 10, and 35 pores per inch (ppi) (Figure 16A). They were also compared with commercial ceria fiber isolation mats (Zircar Zirconia Inc.), which were 7 μm diameter, 100 μm long, and had 88% porosity. DS-RPC foams were also doped with 10 and 20 mol% Zr, to form Ce_0.9_Zr_0.1_O_2_ (CZO-10) and Ce_0.8_Zr_0.2_O_2_ (CZO-20). DS-RPC foams were made with 30 vol% carbon added as a pore forming agent; a 10 ppi SS-RPC foam was also made with no carbon added. The physical characteristics of all of these various materials are shown in Table 1.

**Table 1:** Large (mm-range) and small scale (μm-range) porosity, as well as mass and density, of ceria single scale (SS) and dual scale (DS) RPC foams.

**Sample**	**Large scale porosity (mm-range)**	**Small scale porosity (μm-range)**	**Material**	**Mass (g)**	**Density (g cm^**−3**^)**
8 ppi ceria DS-RPC	8 ppi	18% Ref. 17	CeO_2_	16.42	1.26
10 ppi ceria DS-RPC	10 ppi	18% Ref. 17	CeO_2_	18.41	1.41
35 ppi ceria DS-RPC	35 ppi	18% Ref. 17	CeO_2_	11.63	0.89
10 ppi ceria SS-RPC	10 ppi	none	CeO_2_	27.71	2.12
ceria fiber sample	none	88%	CeO_2_	9.65	0.86
10 ppi CZO_10 DS-RPC	10 ppi	18% Ref. 17	10 mol% Zr doped CeO_2_	16.39	1.26
10 ppi CZO_20 DS-RPC	10 ppi	18% Ref. 17	20 mol% Zr doped CeO_2_	20.45	1.57

“*Ref. 17” in this table is Furler et al. (2014) in this review. From Takacs et al. (2017), used with permission*.

Thermochemical cycling was carried out in a solar TGA powered by a high flux solar simulator. Reduction and oxidation temperatures of ~1,325 and ~825°C were obtained with concentration ratios of C = 1,280 and 440 suns, respectively, in short cycles of 10 min for each step. Single TGA measurements for shorter periods (5 min reduction, 4 min oxidation) are shown in Figures 16B,C, and it was observed that weight change had virtually ceased after these periods. The temperatures shown here are for the bottom of the samples, and show that the fibers did not heat up as much as the foams did, as they created more of a thermal barrier due to their dense structure on the macro (mm) scale despite their much greater micron scale porosity, only reaching ~1,040°C. This is barely enough for reduction to occur. This means that they have a greater optical thickness, making them opaque to incident solar radiation, and diminishing their heating efficiency—an important factor for solar energy materials. Typical mean values of the effective extinction coefficient were found to be 280 m^−1^ for the RPC and 40,000 m^−1^ for the fiber, an optical thickness two orders of magnitude higher for the ceria fibers (Furler et al., 2012b). Nevertheless, this would not have affected the temperature nearer the top surface of the sample, and they still achieved a weight loss of ~2.3 mg cm^−3^ after 5 min, compared to around 4 mg cm^3^ for the best of the DS-RPC foams. The 10, 8 and 35 ppi foams achieved a weight loss of 2.5 mg cm^−3^ after only 1.3, 1.9, and 3.1 min, respectively. The 35 ppi foam with more, but smaller, macropores also reached a slightly lower maximum reduction temperature on the bottom of the sample than the other two foams, indicating a greater optical thickness, and it required 140% more energy to reduce than the 10 ppi RPC foam. As has been discussed above, the macroporosity affects the reduction rate through thermal radiation, but the microporosity in the struts affects the oxidation rates, and all three RPC foams, with broadly similar microporosities and SSAs, had very similar re-oxidation rates (Figure 16C), irrespective of their ppi macropore size (Takacs et al., 2017).

Thermochemical cycles are also shown for the 10 ppi Zr doped DS-RPCs in Figure 16D. As would be expected, the heating profiles were virtually identical, as they have very similar structures, and the addition of Zr increased the degree of reduction in the 1st step, indicated by a greater mass loss, equating to an O_2_ release of 4.2 ml g^−1^ for CZO-20, 3.3 ml g^−1^ for CZO-10, and 2.6 ml g^−1^ for the undoped ceria. However, during subsequent oxidation with CO_2_, the undoped ceria had faster reaction rates, reaching an oxidation extent of 80% to yield 4.4 ml g^−1^ in 9 min. Conversely, CZO-10 and CZO-20 were much slower, reaching only 50 and 25% oxidation after 9 min to produce 2.8 and 1.5 ml g^−1^, respectively, agreeing with previous reports (Scheffe et al., 2013). Due to this incomplete oxidation, the following reduction step was also slower and realized less O_2_ with increasing Zr content. This confirms that pure ceria has more favorable oxidation thermodynamics for the rapid reduction and oxidation cycles used here, negating the advantage of the greater reduction, but slower oxidation, possible with Zr doped ceria (Takacs et al., 2017).

**Figure 17:**
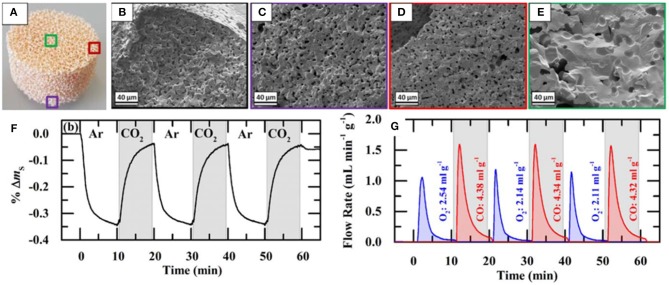
SEM images of the 10 ppi ceria DS-RPC: **(A)** Locations where the SEM images were taken; **(B)** Before redox cycling; **(C)** After redox cycling at the edge of the bottom surface; **(D)** After redox cycling at the edge of the top surface; **(E)** After redox cycling (1,325°C reduction, 875°C oxidation) at the center of the irradiated top surface. Solar-TG measurements for the 10 ppi ceria DS-RPC: mass specific **(F)** % mass change and **(G)** O_2_ and CO evolution rates and total O_2_ and CO evolved, as a function of time during 3 consecutive redox cycles. From Takacs et al. (2017), used with permission.

SEM images of struts of 10 ppi DS-RPC are shown in Figures 17A–E, before and after cycling. Little grain growth was observed on the bottom of the foam (Figure 17C), where the temperature was shown to be much lower (hundreds of degrees less) during solar heating in the reduction step. On the edge of the top surface, a little more sintering and grain growth had occurred (Figure 17D), but in the center of the top surface, where the concentrated solar beam of 1,660 suns was focussed, with temperatures exceeding 1,625°C, a great deal of sintering and grain growth was observed (Figure 17E). Despite this, no significant change in redox was observed over 3 cycles for 10 ppi DS-RPC (Figures 17F,G), with a steady mass change, probably because only a small volume of the foam underwent this extreme sintering. This emphasizes how the experienced heat throughout the porous volume of the reactive material is not even and uniform. Ten ppi DS-RPC achieved peak production rates and total production of ~1.0–1.2 ml min^−1^ g^−1^ and 2.11–2.54 ml g^−1^ for O_2_, and ~1.6 ml min^−1^ g^−1^ and 4.32–4.38 ml g^−1^ for CO. The CO production in all 3 cycles, and O_2_ production in the 2nd and 3rd cycles, was almost constant, and gave a CO:O_2_ ratio of 2.02 and 2.05 for the 2nd and 3rd cycles (Takacs et al., 2017).

**Figure 18:**
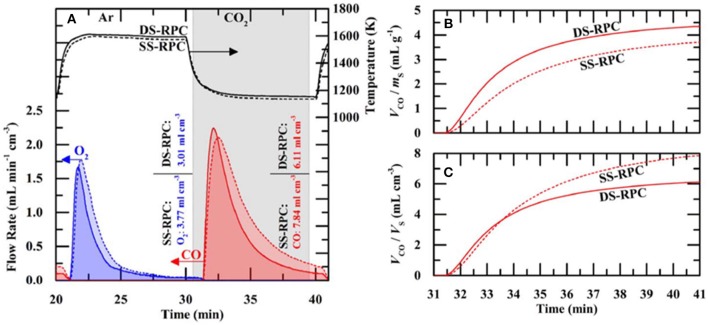
Thermochemical CO_2_ splitting compared for single-scale porosity and dual-scale porosity RPC ceria foams. **(A)** Volume-specific flow rate of O2 and CO and temperature for ceria RPCs with single-scale porosity (SS-RPC) (dashed lines) dual-scale porosity (DS-RPC) (solid lines) during the second redox cycle. Additionally, integrated mass specific O_2_ and CO yields are shown. **(B)** Mass-specific and **(C)** volumes specific CO yield of both RPCs with time during the second oxidation. From Takacs et al. (2017), used with permission.

The influence of the dual porosity on the fuel yield, due to strut porosity, was investigated by comparing the redox performance of 10 ppi SS-RPC (without porous struts) and 10 ppi DS-RPC (with porous struts). The second redox cycle, after stable redox had been established is shown in Figure 18A, and it can be observed that, as expected, the change in microporosity in the struts had little effect on the absorption and transfer of heat radiation, with the DS-RPC experiencing slightly higher temperatures. The peak O_2_ and CO production rates were virtually equal, but SS-RPC consistently showed a slower reaction profile, starting, and ending production after DS-RPC. If absolute quantities are considered (Figure 18A), SS-RPC produced more gasses than DS-RPC, but it is also much denser (2.12 vs. 1.41 g cm^−3^), and an equal volume of each foam was used in the reactor. In terms of specific mass production, DS-RPC was superior to SS-RPC, producing 4.4 ml g^−1^ of CO vs. 3.7 ml g^−1^ for SS-RPC. Due to the larger SSA of DS-RPC with μm-size strut porosity (Furler et al., 2014), it also exhibited faster oxidation rates (Figure 18B), agreeing with experiments carried out in a solar reactor, where the duration of the oxidation step was reduced from 20 min for SS-RPC to only 8 min for DS-RPC (Marxer et al., 2015). Regarding the volume-specific fuel yields, SS-RPC was superior to DS-RPC because of its higher density (Figure 18C; Takacs et al., 2017).

Other investigations have been carried out on normal RPC ceria foams with only 1 level of porosity. In a TGA study on a porous ceria with carbon dioxide splitting at 1,100°C and thermal reduction at 1,450°C, 86% of initial fuel production was retained after 2,000 cycles, and the mean oxygen deficiency value of δ was found to be 0.0197 (Rhodes et al., 2015). Graphite was used as a pore forming agent via formation of CO_2_ bubbles during synthesis. The porous ceria structure was retained for over 2,000 cycles, despite the apparent loss of some surface area, and the oxidation became increasingly homogenous throughout the sample over an increasing number of cycles (Rhodes et al., 2015). Thermochemical modeling showed that for porosities of 0.60, 0.75 and 0.90, the rate of oxygen production, and hence the efficiency of solar-to-chemical energy conversion for CO_2_ (800–1,500°C), increased linearly as the mean pore diameter decreased from 1,000 to 30 μm. For a porosity of 0.90, this continued to increase down to 10 μm, and an energy conversion efficiency of 10.9% was achieved with a modeled solar concentration ratio of C = 3,000 suns at the aperture, but only 120 suns at the surface of the porous structure (Keene et al., 2014).

**Figure 19:**
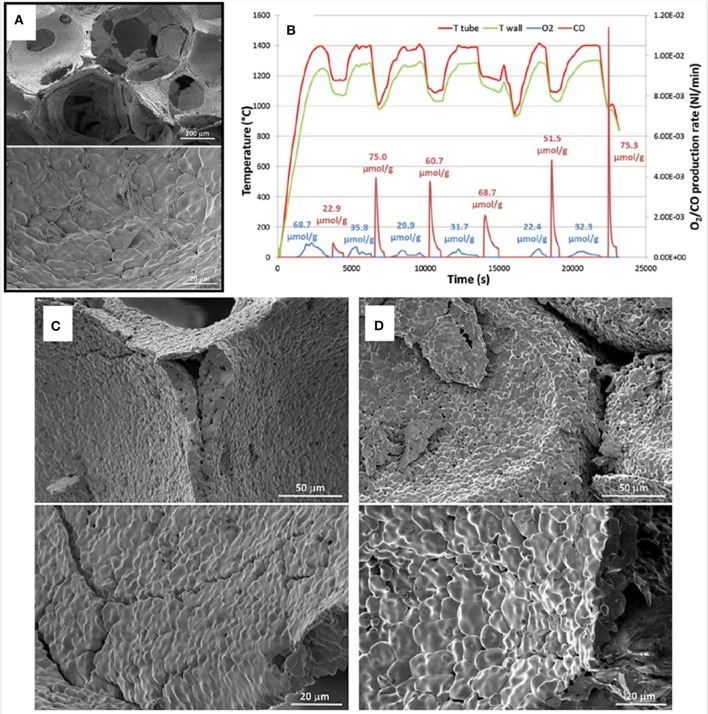
Ceria foam made by replication from a commercial polyurethane foam template with added binder and stabilizers (CF), and its thermochemical splitting of CO_2_. **(A)** SEM micrographs of CF foam after two consecutive TGA with reduction at 1,400°C and oxidation at 1,050°C. **(B)** Solar thermochemical reduction and re-oxidation of CF foam for 6 cycles at ~900 W m^−2^. SEM micrographs of CF foam before **(C)** and after **(D)** 6 solar thermochemical cycles. From Oliveira et al. (2018), used with permission from Elsevier.

Ceria foams were also made by the authors, using the replication from a commercial polyurethane foam template, with a mean cell size of 700 mm = 36 ppi (Oliveira et al., 2018). This was impregnated with a ceria slurry, with (CF) and without (CFBL) added binder and stabilizers, and sintered at 1,450°C to produce the ceria foams. The foams were put through two TGA cycles with reduction at 1,400°C and oxidation at 1,050°C. The foam made with additives (CF) released more O_2_ in the 1st reduction cycle (117 μmol g^−1^ = ~2.64 ml g^−1^), but in the 2nd cycle released exactly the same amount as the foam without additives (CFBL) released in both reduction cycles – 51 μmol g^−1^ (~1.14 ml g^−1^) – indicating that the additives released extra O_2_ as they were reduced in the first cycle. In the oxidation step, CF produced less CO (59 and 84 μmol g^−1^ in cycles one and two, = ~1.32 and ~1.88 ml g^−1^) compared to CFBL (81 and 95 μmol g^−1^, = ~1.81 and ~2.13 ml g^−1^). This was because the additives in CF also produced small secondary phases of α-Al_2_O_3_ and Ce_4.67_Si_3_O_13_ in reactions under reduction, and needle like crystals typical of mullite were also observed in SEM images of CF after cycling, and ceria grains of up to 15 μm were also observed (Figure 19A). These additives were useful to help preserve the structure of the foam, but they had a negative effect on CO production. CF foam had a density of 1.01 g cm^−3^, correlating to a porosity of ~86%.

The CF foam was also run through 6 cycles while heated under actual indirect concentrated solar light, with reduction at 1,400°C/15 min and oxidation between 1,200 and 1,000°C/15 min (Figure 19B). The O_2_ production was greater for the 1st cycle (68.7 μmol g^−1^ = ~1.54 ml g^−1^), but in subsequent cycles it was lower and relatively stable between 22.4 and 35.8 μmol g^−1^ (~0.50–0.80 ml g^−1^), the same trend as observed in the TGA measurements. The 1st cycle showed much lower CO production (22.9 μmol g^−1^ = ~0.51 ml g^−1^), but subsequent cycles had rates of 52–75 μmol g^−1^ (~1.16–1.68 ml g^−1^), depending mainly on the oxidation temperature and the extent of the previous reduction step, with decreasing oxidation temperatures giving faster CO production rates. The foam structure was maintained after the solar cycles, but the ceria grains had grown from 5 to 10–15 μm afterwards (Figures 19C,D). However, the needle-like crystals observed after 2 TGA cycles were not observed, but instead a network of intergranular pockets (<1 μm) had formed at triple joints of grains, consisting of cerium silicate, and occasional 1 μm hexagonal α-Al_2_O_3_ grains on the surface of the larger ceria grains (Oliveira et al., 2018).

## Biomimetic Porous Ceria From Natural Templates

**Figure 20:**
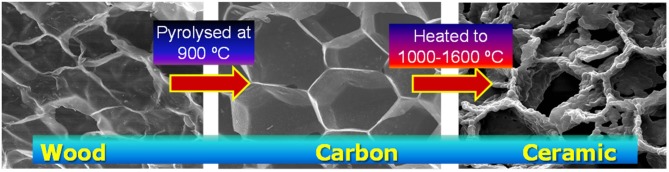
The process of converting wood, in this case cork, to ecoceramic via pyrolysis under N_2_, impregnation, and subsequent heating in air.

Another approach is the use of naturally occurring sustainable materials, e.g., as wood, wood wastes and cork, as templates to create biomorphic/biomimetic ceramics, also known as environmentally conscious ceramics, or ecoceramics (Singh et al., 2003). To make the biomimetic ecoceramic, the wood template is normally firstly pyrolysed in an oxygen-free atmosphere to convert it to carbon. This is then infiltrated with a precursor solution or gas and then heated in air to form the ceramic. The carbon skeleton is lost, but the ceramic takes on the form of the original wood template. The sequence for cork ecoceramics is shown in Figure 20. The phrase ecoceramics, or “environmentally conscious ceramics,” was first coined by Singh et al. at NASA (Singh et al., 2003), and the wood template must come from a sustainable source to qualify.

**Figure 21:**
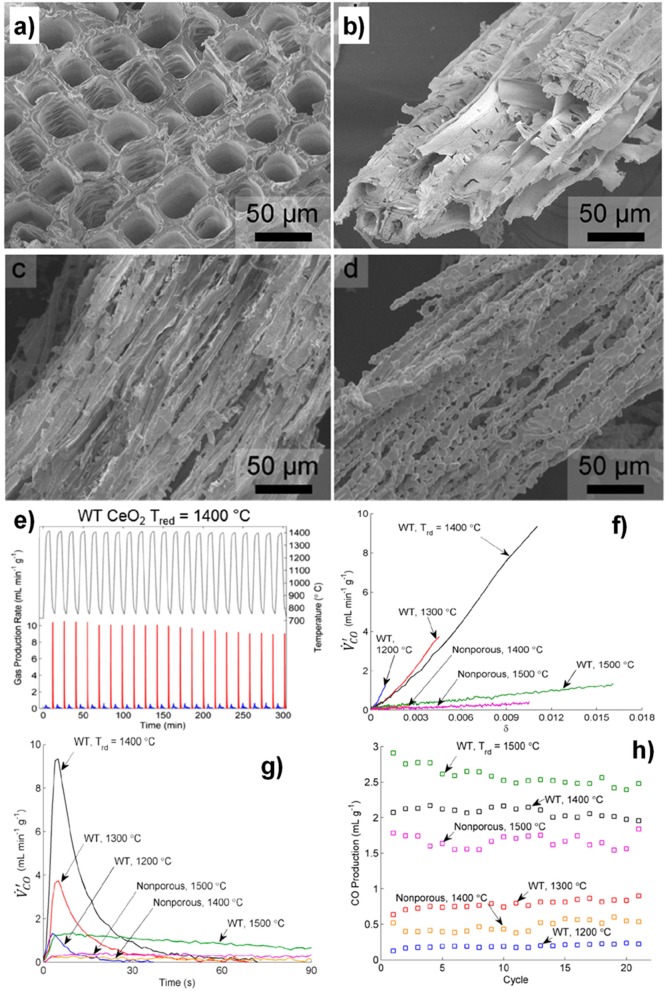
Ceria ecoceramics made from pine wood and their thermochemical splitting of CO_2_ in an infra-red furnace. SEM images of **(a)** pine wood coated with a Ce^3+^ Pechini gel and **(b)** the wood-templated CeO_2_ (WT) product. SEM images of wood templated CeO_2_ (WT) after sintering at **(c)** 1,400°C and **(d)** 1,500°C. **(e)** O_2_ (blue) and CO (red) production rates from WT for 21 cycles at a reduction temperature of 1,400°C. **(f)** CO production rate as a function of δ for WT at 800°C following reduction at 1,200–1,500°C, and for non-porous CeO_2_ at 800°C following reduction at 1,400–1,500°C. **(g)** Change in CO production rate over the first 90 s of oxidation at 800°C for WT following reduction at 1,200–1,500°C, and for non-porous CeO_2_ powder following reduction at 1,400–1,500°C. WT reduced at 1,500°C and both non-porous CeO2 samples continued to produce measurable CO after 90 s (not shown). **(h)** Total CO produced during the oxidation step in each thermochemical cycle for WT and non-porous and CeO_2_ after reduction at the indicated temperatures. Adapted with permission from Malonzo et al. (2014). Copyright 2014 American Chemical Society.

Eastern white pine was used as a template to produce ceria ecoceramics in 2014 (Malonzo et al., 2014). In this case, 4 × 5 × 0.5 mm sections of pine wood were soaked in a Pechini synthesis precursor solution, the solution gelled in the wood at 90°C, and then heated to 310°C for the combustion synthesis to occur, followed by sintering at 1,200–1,500°C, all in air. No pyrolysis step was used in this process. The pine wood had 20 μm diameter elongated rectangular pores, with 10 μm wide cell walls (Figures 21a,b). However, when it was converted to an ecoceramic, a large quantity of the wood template was lost, creating a fragile material still with 20 μm cells, but with cell walls of only 1 μm thickness. When sintered at 1,400°C the ceria ecoceramic only had a surface area of 0.1 m^2^ g^−1^, which reduced further to <0.05 m^2^ g^−1^ at 1,500°C, as extensive grain growth occurred (Figures 21c,d). Nevertheless, the ceria retained much of the microstructure of the wood template, with an open porous structure, and XRD showed it to be pure cubic CeO_2_.

The pine wood-templated CeO_2_ (WT) was sieved to leave particles between 180 and 850 μm, and heated in an infra-red furnace, being reduced at 1,200–1,500°C/15 min, and then oxidized with CO_2_ at 800°C/ 15 min, for 21 cycles. Non-porous ceria powder was cycled for a comparison. WT exhibited quite regular and repeatable cycles at all reduction temperatures, but with greatly varying yields—see Figure 21e for an example of 21 WT cycles at 1,400°C. At 1,200°C, very little reduction occurred with an estimated δ for CeO_2−δ_ of only ~0.001, increasing to ~0.0045 at 1,300°C, 0.012 at 1,400°C and 0.0165 at 1,500°C (Figure 21f). These values were much greater than the standard ceria powder achieved at 1,400 and 1,500°C (actual O_2_ production quantities were not given).

The change of rate of CO production with δ can also be seen in Figure 21f, the rate of oxidation for WT being 5–6 times faster that of the non-porous CeO2 after reduction at 1,400°C, and 2–3 times faster after reduction at 1,500°C. The rate of production peaked for WT reduced at 1,400°C, despite the fact that a higher δ was achieved at 1,500°C, probably due to the loss of porosity at the higher temperature. The cells were found to collapse with thermal cycling at 1,500°C as grain growth caused the cell walls to fuse together, reducing the active SSA, and decreasing the CO production rate by a factor of 7 compared to that seen after oxidation after reduction at 1,400°C (falling from 9.5 to 1.3 ml min^−1^ g^−1^). The peak rate for the poorly reduced but highly porous WT reduced at 1,200°C was identical to that of the well reduced but poorly porous WT reduced at 1,500°C. Although the rate clearly peaked for WT when reduced at 1,400°C, the gradient of V'_CO_ vs. δ was greatest at 1,300 for WT reduced at 1,200°C, with an equal gradient of 800 for WT reduced at 1,300 and 1,400°C, and very low gradients of <70 for WT reduced at 1,500°C and standard ceria powder reduced at 1,400 and 1,500°C. This indicates that both the degree of reduction (greater at higher temperatures) and preservation of the open porous structure and surface area (greater at lower temperatures) are important, and for the best results a compromise must be achieved between the two, in this case for WT reduced at 1,400°C (Malonzo et al., 2014).

Variations of the CO production rate with time are shown in Figure 21g, and it can be observed that while WT reduced at 1,400°C clearly had the greatest peak rate, all three WT samples up to that temperature reached their peak rate very rapidly after a few seconds, and had virtually stopped CO production after 30–40 s. By comparison, the non-porous ceria, and WT reduced at 1,500°C which had greatly compromised porosity and surface area, exhibited low but constant peak production rates throughout the 90 s period. This demonstrates how the porous structure of WT greatly enhances the speed of initial oxidation in CO_2._

The total amount of CO produced in each of the 21 cycles is shown in Figure 21h, and it can be seen that the values are reasonably regular throughout the cycles. Interestingly, although WT reduced at 1,500°C had a much lower CO production rate, the fact that it maintained a steady production for at least 90 s, and continued to generate some CO throughout the entire 15 min of oxidation, meant that it actually had the largest CO production of ~2.5 ml g^−1^, compared to ~1.7 ml g^−1^ for non-porous ceria reduced at 1,500°C, ~2.1 ml g^−1^ for WT reduced at 1,400°C, and ~0.5 ml g^−1^ for non-porous ceria reduced at 1,400°C. This meant that WT produced 4 times more CO than non-porous ceria powder when both were reduced at 1,400°C, and 1.5 times more when both were reduced at 1,500°C (Malonzo et al., 2014).

Pullar et al. have carried out innovative work to create biomorphic ecoceramics of ferrites and ceria from cork templates (Pullar et al., 2015, 2016; Pullar and Novais, 2017). Cork naturally has similar dimensions to the pine-derived ecoceramic above, yet with a more regular 3-DOM (three-dimensionally ordered macroporous) microstructure. Cork has a very porous 3-DOM microstructure made of elongated hexagonal cells which are ~15–20 μm in diameter and 40–50 μm in length (Gil, 2014). These cells have walls only around 1 μm thick, giving an extremely porous, light-weight and regular microstructure, which can contain up to 200 million cells per cm^3^ (Pereira, 2007). Therefore, it would appear to be an ideal template material. Cork is the bark of *Quercus suber L*., an evergreen oak tree found around the Mediterranean basin (Portugal supplying circa 50% of global cork production; APCOR, 2016). The cork is harvested without harming the tree, which lives on as a carbon sink, with a productive life of around 200 years. The cork bark regenerates after ~10 years, to be harvested again, and absorbs the equivalent amount of CO_2_ which could be released from the cork during processing or combustion. Cork forests sequester up to 5.7 T CO_2_/ha/yr (Gil, 2014). As such, cork is a truly renewable and sustainable resource.

**Figure 22:**
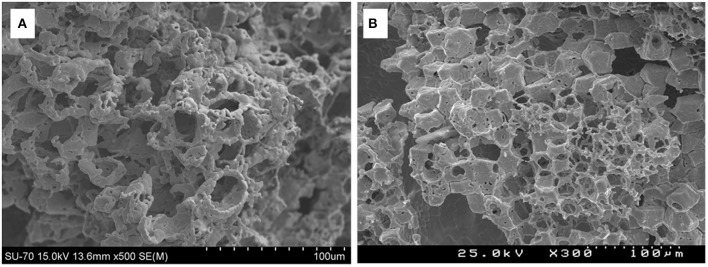
SEM micrographs of cork-derived ceria ecoceramics (CG), depicting their cork-like structure after synthesis at 1,600°C **(A)**. Note that some of the cork cells in **(B)** have been completely filled with ceria during processing, resulting in an “inverse” cork structure.

The authors have recently reported the successful splitting of CO_2_ using cork-derived ceria ecoceramics (Oliveira et al., 2018). Cork-template-based ceria granules (CG) were synthesized from cork granules (several millimeters in size) via pyrolysis under N_2_ at 900°C/30 min to form the carbon template, which was then infiltrated with cerium nitrate solution. After drying, this was calcined in air at 1,600°C/30 min to remove the carbon, creating a pure CeO_2_ ceramic which preserved the 3-DOM cork microstructure. After heating to 1,600°C, CG had a low density of only 0.41 g cm^−3^ (~6% of the maximum theoretical density of ceria), equivalent to a large porosity of ~94%, although the surface area was <1 m^2^ g^−1^, a virtually unavoidable problem at such high-temperatures where materials sintering is unavoidable. XRD showed CG to be pure single phase crystalline ceria, with the microstructure shown below in Figure 22A. CG had mostly maintained a cork-like microstructure, with ~20 μm diameter cells and quite thin cell walls, although grain growth had occurred during the heat treatment at 1,600°C. In much of the ceria ecoceramic, an “inverse” cork structure had formed, where the ceria was in dense sections with the hexagonal form of the cells, separated by gaps equivalent to the original cell walls, and Figure 22B shows that within the same mm sized granule of CG, both the porous cork microstructure and the non-porous “inverse” cork structure co-exist. In the future, we will endeavor to avoid this “inverse” cork structure, which should inevitably result in even greater enhancement of its redox capabilities for CO_2_ splitting.

During laboratory (i.e., non-solar) thermochemical cycling of CG, it was found that the reduction began at ~1,030°C (with a sharp mass loss), and the reaction rate increased rapidly with increasing temperature. The peak O_2_ production rate was 0.06 μmol s^−1^ g^−1^ (~0.08 ml min^−1^ g^−1^) when nearing 1,400°C, although as the reduction is thought to be a thermally activated process, proceeding with continuous oxygen loss from the ceria lattice as temperature increases, the reduction rate immediately decreased as the temperature ceased rising. When cooled to 1,050°C in Ar, the reduction stopped and the mass was stable. When CO_2_ was injected, this caused a sudden mass increase due to the rapid incorporation of oxygen into the ceria lattice, with a peak CO production rate of 0.59 μmol s^−1^ g^−1^ (~0.79 ml min^−1^ g^−1^, 10 times greater than the peak O_2_ production rate). Therefore, the oxidation reaction was significantly faster than that of reduction, agreeing with findings for other cerias (Le Gal et al., 2011; Furler et al., 2014). During the reduction step at 1,400°C, the quantity of oxygen released was 51 μmol g^−1^ (~1.14 ml g^−1^), giving a reduction yield of 3.5%, which also agrees with data reported previously (Le Gal et al., 2011). The maximum mass loss for CG corresponded to a non-stoichiometric δ value of 0.03 (CeO_2_ → CeO_2−δ_). Whatever microstructure or morphology it has, the thermal reduction step is the main limiting factor in the ceria redox cycle, as pure ceria is difficult to reduce to high δ values (δ is usually limited to around 0.02–0.05, unless extremely low pressures or high temperatures are used), but is easily oxidized (Rhodes et al., 2015).

**Figure 23:**
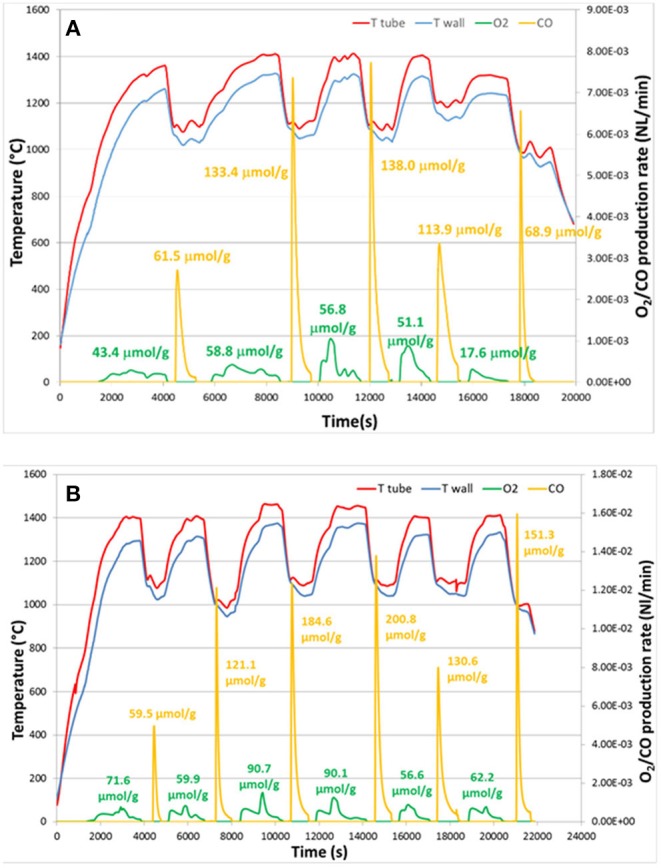
Time-dependent solar thermochemical reduction and re-oxidation profiles of ceria ecoceramic granules with cork structure (CG) for 1st series of 5 cycles at DNI~750 W m^−2^ **(A)**, and 2nd series of 6 cycles at DNI~1,000 W m^−2^ **(B)**. From Oliveira et al. (2018), used with permission from Elsevier.

The redox performance of CG was then investigated in a solar reactor (medium scale solar furnace, MSSF) at PROMES-CNRS under real solar irradiation conditions. Evolution rates of O_2_ and CO and the temperatures inside the reaction vessel (T_tube_) and on the cavity external wall (T_wall_) are shown in Figure 23. The reduction step occurred at about 1,400°C, controlled by opening/closing a shutter according to dynamic variations of the direct normal irradiance (DNI). The oxidation temperature was varied from one cycle to another in the range of 1,000–1,200°C, and significantly affected the amount of CO produced. The thermochemical redox cycling was carried with a temperature swing, alternatively switching between an Ar atmosphere for reduction, and a CO_2_ atmosphere (50% in Ar) for oxidation (Oliveira et al., 2018).

A total of 11 thermochemical redox cycles were performed, as shown in Figures 23A,B, in two different series. The first series of 5 cycles was performed with a lower DNI of ~750 W m^−2^ (Figure 23A), with maximum temperatures of only 1,360°C during the 1st cycle and 1,320°C during the last (5th) cycle. This resulted in a lower reduction extent than that at ~1,400°C in the 2nd, 3^rd^, and 4th cycles. The quantity of CO production was directly related to the reduction step temperature—the higher the temperature of reduction, the greater the amount of CO produced. The amount of O_2_ produced in the 4th cycle at 1,400°C was similar to the laboratory measurements (~51 μmol g^−1^ = ~1.14 ml g^−1^), and a slight temperature increase above 1,400°C increased O_2_ production in the 2nd and 3rd cycles. Variations in the oxidation temperature greatly influenced the CO production rate, with the peak of CO production becoming greatly broadened to a slower rate over a longer period, and with less CO produced, when increasing the oxidation temperature to 1,200°C (113.9 μmol g^−1^ = ~2.55 ml g^−1^ for the 4th cycle at 1,200°C, vs. 138.0 μmol g^−1^ = ~3.09 ml g^−1^ for the 3rd cycle at 1,100°C). When the oxidation temperature was lower at only 1,000°C in the 5th cycle, an even narrower peak was seen, equal to a production of only 68.9 μmol g^−1^ (~1.54 ml g^−1^), because of the lower reduction temperature at 1,320°C. This means that when decreasing the oxidation temperature below 1,200°C the peak CO production rate increased, while the period of oxidation was shortened (as shown by the sharper CO production peak).

The second series of six cycles was performed at a higher DNI of ~1,000 W m^−2^ (Figure 23B), increasing the reduction temperature up to 1,450°C in the 3rd and 4th cycles. This had a large impact on the reduction yield, with O_2_ production reaching over 90 μmol g^−1^ (2.016 ml g^−1^), a 50% increase of the extent of reduction (δ) when compared to 1,400°C in the 2nd, 5th, and 6th cycles (~60 μmol g^−1^ = ~1.34 ml g^−1^). A peak O_2_ production rate of 0.13 μmol s^−1^ g^−1^ (~0.175 ml min^−1^ g^−1^) was also seen in the 3rd cycle, which led to an increased maximum CO production yield. The peak CO production rate was up to 1.43 μmol s^−1^ g^−1^ (~1.92 ml min^−1^ g^−1^) in the 6th cycle performed at an oxidation temperature of only 1,000°C. This was compared to the best CO production rate at 1,400°C of 1.26 μmol s^−1^ g^−1^ (~1.69 ml min^−1^ g^−1^) in the 2nd cycle. Altogether, this demonstrated the cycling stability of CG under real indirect solar irradiation conditions. Reduction temperature strongly influenced the extent of reduction and the total yield of CO produced, while the oxidation temperature had more effect on the rate of CO production (Oliveira et al., 2018).

The reaction rate was also directly related to the temperature, and increasing reduction temperatures by only 50°C (from 1,400 to 1,450°C) increased O_2_ yield from 60 to 90 μmol g^−1^ (= ~1.34–2.02 ml g^−1^). Consequently, the yield of CO was in the range of 185–200 μmol g^−1^ (= ~4.14–4.48 ml g^−1^), greater than that observed at 1,400°C, which was in the range of 120–150 μmol g^−1^ (= ~2.69–3.36 ml g^−1^). This corresponds to an increase of ~35–55%. The kinetics of oxidation was also made faster by lowering the temperature to 1,000°C, showing that the oxidation was favored by decreasing the temperature (effectively increasing the temperature gap between the redox steps), which agrees with thermodynamic predictions. As an example, the last cycle, performed with oxidation at 1,000°C demonstrated the highest CO production rate. The oxidation reaction was significantly faster than reduction, with CO production rates showing a very sharp peak as soon as the CO_2_ was injected, CO production then decreasing steadily, with a total oxidation peak of around 10–15 min. In contrast, O_2_ production rates were highly sensitive to small variations in temperature, featuring a broadened pattern that fluctuated in response to small temperature changes. The total period of O_2_ release was around 30 min, including both heating up time to the maximum temperature and the dwell time at this temperature. The reduction was immediately triggered when the CO_2_ injection was stopped, initiating the release of O_2_ in greater quantities as the temperature was increased, but the O_2_ release evolved smoothly (Oliveira et al., 2018).

To summarize, cork templated ceria ecoceramics (CG) showed very repeatable cycling, producing ~130 μmol g^−1^ (~2.91 ml g^−1^) CO with reduction at 1,400°C, and ~200 μmol g^−1^ (~4.48 ml g^−1^) CO at 1,450°C. The very high CO production rate demonstrated the advantage of the 3-DOM cork structure, and CO production stability was maintained during cycling, demonstrating that the morphology of CG positively affected the redox performance.

As discussed already, porous structures are known to favor the oxidation step due to their high SSAs (Furler et al., 2014). CG produced cumulative amounts of 659 μmol g^−1^ (~14.8 ml g^−1^) of O_2_ and 1,364 μmol g^−1^ (~30.5 ml g^−1^) of CO over 11 cycles, giving a total CO/O_2_ ratio of about 2, equal to the theoretical ratio. The redox activity and CO_2_ splitting capability of the cork-templated CG ceria was better than that of the artificial porous ceria foam under the same conditions (section 6) (Oliveira et al., 2018). These CO yields from the artificial foam corresponded to less than half of that from CG under the same conditions, and the CO fuel production rate was almost three times lower than that obtained with CG at lower reduction temperatures, while the total specific CO release was similar (around ~4.8 ml g^−1^). For a ceria RPC foam with dual-scale porosity (Marxer et al., 2015) the peak O_2_ production rate was 0.19 ml min^−1^ g^−1^, similar to that of CG, but the peak CO release rate was much slower at 0.75 ml min^−1^ g^−1^, even though a reduction step was used over 1,500°C (Chuayboon et al., 2019).

The observed reaction rate enhancement of a factor of two for CG compared to artificial ceria foam might be attributed to the fact that the cell size of CG is at least one order of magnitude smaller. The extent of reaction extent is not only limited by the thermodynamics (such as temperature and oxygen partial pressure), but also by other factors such as thermal and mass transport. Such transport properties are affected by physical material properties (thermal diffusivity, thermal conductivity), as well as morphology (size and shape of pores). These results demonstrate the superior reactivity of this cork-structured ceria CG, compared with porous ceria foams with either single-scale (Furler et al., 2012b; Oliveira et al., 2018) or dual-scale porosity (Furler et al., 2014).

## Summary

As this review clearly demonstrates, as well as grain size, stoichiometry and effects of dopants, variations in porosity and microstructure can also have a great effect on the ability of ceria to split CO_2_. It is very difficult to directly compare results and attribute differences purely to microstructure, as there are many other variables between the CO_2_ splitting experiments of different research groups, such as gas partial pressures, heating rates and dwell times, source of heating energy (solar, electrical, IR, TGA), quantity and form of sample, etc. Nevertheless, in this summary we will attempt to compare and summarize the key results in this review regarding the effects of microstructure and porosity on thermochemical CO_2_ splitting by ceria, as detailed in Table 2.

**Table 2:** Effect of ceria morphology and microstructure on thermochemical CO_2_ splitting, along with the redox reaction conditions used.

**Type of microstructure**	**Energy source**			**Thermal reduction temperature (^**°**^C)**	**Oxidation temperature (^**°**^C)**	**Degree of non-stoichiometry (δ)**	**O**_****2****_ **evolution rate (ml min**^****−1****^ **g**^****−1****^**)**		**Total O_**2**_ yield (ml g^**−1**^)**	**CO evolution rate (ml min**^****−1****^ **g**^****−1****^**)**		**Total CO yield (ml g^**−1**^)**	**Solar to fuel efficiency (%)**	**References**
	**Conc. solar light**	**Electrical heating**					**peak**	**average**		**peak**	**average**			
		**TGA**	**Electric or IR furnaces**											
Ceria powder		X		1,400	1,000	0.037	0.028	0.026	1.19	0.067	0.065	2.28	–	Le Gal et al., 2011
Ceria powder		X		1,500	1,000	0.088	0.029	0.028	2.78	0.033	0.031	5.47	–	Bonk et al., 2015
Ceria powder			X	1,500	1,000	–	0.141	0.136	4.23	0.114	0.113	5.01	–	Zhu et al., 2018
Non-porous bulk ceria			X	1,400	800	0.0027	0.40	–	–	0.35	0.03	0.5	–	Malonzo et al., 2014
				1,500	800	0.0105	0.35	–	–	0.50	0.12	1.8	–	
Porous monolithic ceria^$^	X			1,581–1,624	800	–	0.105	0.049	2.74	4.615	1.040	4.31	0.4	Chueh et al., 2010
Porous ceria felt^#^	X^*^			1,650	1,000	0.044	0.20	0.116	2.89	0.6	0.146	2.19	0.15	Furler et al., 2012a
Porous ceria felt	X^*^			1,530	1,200 to 300	–	0.18	0.076	2.66	2.85	0.385	5.39	–	Furler et al., 2012b
Ceria particle fibers			X	1,500	800	0.04	–	0.150	0.75	40.80	0.288	1.15	–	Gladen and Davidson, 2016
Ceria fibers			X	1,200	800	0.0028				4.5		0.40	–	Gibbons et al., 2014
2.5% Zr-doped ceria fibers			X	1,400	800	0.027	0.75	0.4	2.0	43	0.8	3.9	–	
3-DOM ceria powder^%^			X	Isothermal process at 800**°**C, with chemical reduction by 5% H_2_ gas, oxidation with CO_2_ gas		–	–	–	–	36.7	22	22	–	–
Random ceria foam^%^						–	–	–	13.0	6.8	23	–		
Commercial ceria (mm scale)^%^						–	–	–	10.3	7.7	23	–		
3-DOM ceria powder			X^@^	1200	850	–	–	–	–	–	0.83	0.9	–	Rudisill et al., 2013
Non-ordered macroporous powder						–	–	–	–	–	0.50	1.0	–	
D-3DOM (aggregated 3DOM particles)						–	–	–	–	–	0.03	0.08	–	
Non-porous commercial ceria pellet						–	–	–	–	–	0.03	0.06	–	
RPC Reticulated porous ceria foam	X^*^			1,420/22 min	Cooling from 1,200 to 300**°**C/40 min	0.016	0.078	0.044	1.056	0.127	0.043	1.465	0.73	Furler et al., 2012b
				1,530/22 min		0.031	0.163	0.083	2.010	0.365	0.126	4.107	1.44	
				1,600/22 min		0.042	0.216	0.133	2.764	0.526	0.158	5.690	1.73	
RPC-0 (0 vol% pore forming agent)		X		900	1,500	0.037	–	–	–	–	0.22^£^	–	–	Furler et al., 2014
RPC-10 (10% pore forming agent)						–	–	–	–	–	0.31^£^	–	–	
RPC-20 (20% pore forming agent)						–	–	–	–	–	0.40^£^	–	–	
RPC-30 (30% pore forming agent)						–	–	–	–	–	1.12^£^	–	–	
RPC-50 (50% pore forming agent)						0.038	–	2.01	–	–	2.22^£^	4.47		
RPC-30 (30% pore forming agent)	X^*^			1,000–1,575	825–1,300	0.039	0.27	0.17	2.52	1.13	0.63	5.02	1.72	
RPC-30 (30% pore forming agent)	X^*^			1,450–1,500^a^	700–1,000	–	0.4	–	–	1.4	0.7	5	5.25	Marxer et al., 2017
10 ppi RPC SS single scale porosity ceria		X		1,325/10 min	825/10 min	–	0.66	0.18	1.77	1.06	0.37	3.7	–	Takacs et al., 2017
10 ppi RPC DS dual scale porosity ceria							1.2	0.25	2.54	1.6	0.44	4.38		
10 ppi RPC DS 10 mol% Zr ceria							–	0.14	1.4	–	0.28	2.8		
10 ppi RPC DS 20 mol% Zr ceria							–	0.09	0.9	–	0.15	1.5		
Ceria foam	X			1,400	1,000	–	0.054	0.020	0.78	1.32	0.112	1.68	–	Oliveira et al., 2018
Pine wood-templated ceria			X	1,400	800	0.012	–	–	–	9.5	0.140	2.1	–	Malonzo et al., 2014
				1,500	800	0.0165	–	–	–	1.3	0.167	2.5		
Cork-templated 3DOM ceria	X			1,400	1000	0.03	0.100	0.034	1.34	1.69	0.224	3.36	–	Oliveira et al., 2018
				1,450	1,000	–	0.175	0.050	2.02	1.92	0.299	4.48		

*The different colored bands are used to separate these results into those for powder, bulk, fibres, 3-DOM, RPC and biomimetic ecoceramics*.

* *High flux simulated solar light*.

$ *These values are for the first cycle—they dropped by ~30% with subsequent cycles*.

# *CO produced from syngas, not CO_2_*.

% *Extremely high degree of oxygen non-stoichiometry achieved due to H_2_ use in isothermal reduction step, giving very high CO yields*.

@ *Very rapid IR furnace, total cycle = 3 min, reduction = 78 s, oxidation = 90 s, these data after 30 cycles*.

£ *These “average” values were only over the time needed to reach 90% of the total of CO produced, which took 3 min for RPC-50 and 28 min for RPC-0. All samples were actually reduced for a full 60 min in the experiments, so these values cannot really be directly compared to others*.

a *Combined temperature and pressure swing process, with reduction at a pressure of only 10 mbar, oxidation at 1 bar*.

Various ceria powders were investigated by TGA or electric tube furnaces, and they all gave very high CO yields of between 2.28 and 5.47 ml g^−1^ and O_2_ yields of 1.19–4.23 ml g^−1^ (see Table 2; Le Gal et al., 2011; Bonk et al., 2015; Zhu et al., 2018), with rates largely determined by available surface area. However, such powders undergo severe sintering at the high temperatures required, and especially with repeated cycles. Furthermore, these were just loosely packed powders in a laboratory set-up, not really an appropriate form for actual solar CO_2_ splitting applications, which requires large porous monoliths, or sections of at least mm-to cm-scale pieces. Therefore, there is a need for larger scale porous ceria ceramics with similar capabilities. Indeed, non-porous bulk ceria was shown to have low peak and average CO production rates due to a lack of surface area, and required a period of 15 min with a temperature swing of 700°C and reduction at 1,500°C (measured by TGA) to achieve a total CO yield of 1.8 ml g^−1^ (Malonzo et al., 2014), and others reporting even worse production rates and yields (Rudisill et al., 2013).

The porous monolithic ceria used for the first reported thermochemical CO_2_ splitting was actually a very good material, with high total O_2_ and CO yields and CO peak production rate (see Table 2; Chueh et al., 2010), and was achieved with real concentrated solar radiation. However, they used a low oxidation and high reduction temperatures, with a large temperature swing of ~800°C which helped give such high yields, and although the oxidation step was rapid at 4 min (hence the high CO average rate), they had a slow reduction step of 50 min.

Porous ceria felt, made from a sheet of compressed ceria fibers, has been investigated using simulated concentrated solar light, and although high O_2_ and CO yields of 2.66–2.89 and 2.19–5.30 ml g^−1^, respectively, were reported (Furler et al., 2012b,b). In one of these studies the production rates were very low and a high reduction temperature of 1,650°C was required, as the material had a low rate of heat transfer, and the experiment was carried out in a syngas mixture containing both H_2_O and CO_2_ (Furler et al., 2012a), and concentrated solar light was found to destroy the fibrous structure where it was concentrated (Takacs et al., 2017). Another compressed mat of ceria fibers was also found to have a very high CO peak production rate and CO yield of 2.85 ml min^−1^ g^−1^ and 5.39 ml g^−1^, respectively, although they were shown to be poor at thermal conduction away from the exposed surface, due their restively high density (Furler et al., 2012b). Commercial ceria fibers (which in reality were more like needles, with a low aspect ratio) were found to have the 2nd highest CO production rates reported here of 40.8 ml min^−1^ g^−1^ with a temperature swing of 700°C (Gladen and Davidson, 2016), but the average rate was only 0.288 ml min^−1^ g^−1^ throughout the oxidation cycle, and the CO yield was in the lower range of only 1.15 ml g^−1^. This relatively poor yield, combined with the high cost of these fibers, would greatly limit their usefulness. Other pure ceria fibers reported here had poor CO production values, although the same authors reported 2.5 mol% Zr doped fibers with a very high peak and total CO production of 43 ml min^−1^ g^−1^ and 3.9 ml g^−1^, respectively (Gibbons et al., 2014), although this high peak value was reached after only 5 s and then decreased rapidly within 30 s. However, with this low level of Zr doping only leading to slightly reduced oxidation and average CO production rates (0.8 ml min^−1^ g^−1^) compared to the undoped ceria, but with much greater overall yields without requiring the much longer oxidation cycle periods usually required for Zr doped ceria, this appears to be a very promising form of fibrous ceria. In general, the results in Table 2 indicate that ceria fibers are worth further exploration as a potential form of thermochemical reactive ceria.

The 3-DOM ceria reported to date, while undeniably interesting and with great potential, so far has exhibited relatively poor CO production rates and yields (Table 2) (Rudisill et al., 2013). The exception to this was the extremely high CO production yield of 22 ml g^−1^ and peak production rate of 36.7 ml min^−1^ g^−1^ achieved via an isothermal process with chemical reduction by H_2_ of 3-DOM ceria (Venstrom et al., 2011, 2012), as opposed to thermal reduction. However, this cannot really be compared to thermally driven (ideally solar) processes. Indeed, the random ceria foam and mm scale commercial ceria pellets used for comparison in this work had almost identical CO yields of 23 ml g^−1^, indicating it was the reduction process, rather than the 3-DOM nature of the material, that made the greatest contribution to these results.

Reticulated porous ceria (RPC) foam, made using a template of polymer spheres, has been the most greatly studied form of porous ceria for thermochemical CO_2_ splitting, and with very good results, as can be seen in Table 2. These typically have densities below 2 g cm^3^. Using simulated concentrated solar light, total CO yields of 5.02–5.69 ml g-^1^ have been achieved, although the CO production rates tend to be low, <1 ml min^−1^ g^−1^ (Furler et al., 2012b; Marxer et al., 2017; Oliveira et al., 2018). Other studies using electrical heating have shown similar results, even with added porosity from pore forming agents or dual scale porosity from microporous connecting struts (Furler et al., 2014; Takacs et al., 2017). Zr doping appeared to make these RPC foams worse, due to slower, and hence incomplete, oxidation, and an increased degree of sintering and density removing micropores from the struts. However, the Zr-free RPC foams with 30% pore forming agent have shown the highest fuel efficiency values yet reported for porous or 3D structured materials, with an efficiency of 5.25% achieved for a dual scale porosity RPC foam in a 2nd generation simulated solar furnace with improved heat and mass transfer characteristics (Marxer et al., 2017). Although structures with submicron-sized pores exhibited relatively fast oxidation rates, they lacked morphological stability as sintering occurs at elevated temperatures (Venstrom et al., 2012). Furthermore, optically thick structures inhibited penetration of concentrated solar irradiation, resulting in non-uniform heating (Furler et al., 2012b). Hence, further optimisation of the RPC structure through proper selection of the porosity and pore size distribution is required to achieve efficient volumetric radiative absorption and uniform temperature distribution.

The most recent technology utilized to make porous 3-DOM ceria is as a biomimetic ceramic made from natural wood templates, ideally wastes, and these are also known as ecoceramics. Pine wood has been used as a template for porous ceria ecoceramics, but after sintering at 1,400–1,500°C they had greatly reduced surface areas. Nevertheless, it maintained a wood-like microstructure, and when reduced at 1,400°C had a very high peak CO production rate of 9.5 ml min^−1^ g^−1^, probably due to a greater surface area than when reduced at 1,500°C, although the average CO rates and total CO yields were similar when reduced at 1,400 or 1,500°C (Table 2; Malonzo et al., 2014). The actual CO yields achieved were reasonable at 2.1–2.5 ml g^−1^, although inferior to RPCs or some ceria fiber materials. Cork templated ceria ecoceramics have also been reported, and these had superior CO yields of 3.36 and 4.48 ml g^−1^ with temperature swings of only 400 and 450°C, respectively (Oliveira et al., 2018), this upper value being ~80% of the best thermally reduced CO production yields reported for ceria. This was despite the cork derived ceria being sintered at an even higher temperature of 1,600°C, and these tests were carried out using real indirect concentrated solar light. Despite this high sintering temperature, the apparent density of the cork-derived ceria ecoceramics was only 0.41 g cm^−3^, indicating a total porosity of ~94% (Novais and Pullar, 2019). The peak CO production rates for these pine and cork ceria ecoceramics are the highest reported for any 3D/3-DOM porous cerias, and are bested only by some of the fibrous cerias (excluding the chemically reduced ceria RPC), which are much denser materials as they are prepared as fibrous mats. This improvement compared to RPC foams maybe due to the fact the cells in the ecoceramics made from wood are only around 40 μm (from pine) and 20 μm (from cork), compared to the much larger pores found in RPC foams, which range from hundreds to thousands of microns in diameter.

The reported results have demonstrated that the material morphology clearly affects the oxidation kinetics, as well as the heat and mas transfer rates within the reactive porous structure. Identification of proper materials shaping strategies favoring kinetics, while offering suitable redox activity, high oxygen transport properties, and long-term cycling stability under concentrated solar irradiation, is thus required. Relevant morphologies such as 3D porous structures capable of absorbing concentrated solar radiation, while offering a large available geometrical surface area for the solid/gas reactions, are promising options. Using bio-based templating materials and a biomimetic approach to elaborate ecoceramics has to be pursued to advance sustainable processes.

## Future Prospects

In general, CO_2_ could soon become a feedstock of zero, or even negative, cost for conversion to fuel, due to the large amounts of low-cost and relatively pure CO_2_ which is becoming available from current and planned carbon sequestration and storage (CSS) plants (Centi and Perathoner, 2009). In 2018 there are currently 43 large-scale CSS facilities planned or in operation globally (18 in commercial operation, 5 under construction and 20 in various stages of development), which captured 40 Mt of CO_2_ in 2018 (Global CSS Institute, 2018). Apart from positive commercial image and public concerns about climate change, another factor which is driving interest in CO_2_ conversion and use is the existence of many emissions for which CSS options are not possible. It is estimated that ~5–10% of total man-made CO_2_ emissions, which were 30 Gt worldwide in 2008 (International Energy Agency (IEA), 2008) and in a decade have increased to 37 Gt by 2018 (Le Quéré et al., 2018), could be suited for production of fuels (Centi et al., 2008; Holmen, 2009), and solar fuel production is an obviously preferable route.

Development of further innovative processing and synthesis techniques to provide 3-DOM and porous ceria microstructured materials need to be explored. Mesoporous ceria nanoparticles have recently been synthesized through an aqueous one-pot hydrothermal sol-gel route, using hexadecyl-trimethyl-ammonium bromide (CTAB) as a surfactant (Azmi et al., 2019). These ceria nanopowders had a surface area of 28–76 m^2^ g^−1^, albeit when calcined at only 400–500°C, but they showed a CO_2_ uptake of ~200 μmol g^−1^ at a pressure of 1.15 atm, showing that the CO_2_ could diffuse easily and rapidly through them. Such materials could be used as a basis for the creation of novel 3D porous structures.

**Figure 24:**
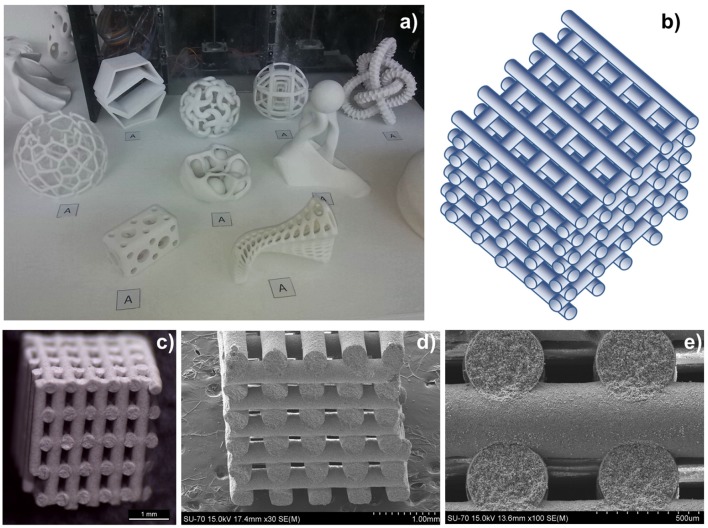
Examples of 3D printing (robocasting) of ceramics. **(a)** Sintered 3D printed porous ceramic structures manufactured at Aveiro University. **(b)** Section of a typical robocast porous bone scaffold structure. **(c–e)** Optical and SEM images of robocast and sintered bioglass scaffolds produced at Aveiro University, with diameters of 400 μm for the ceramic part, and 300 μm for the void.

One intriguing avenue of exploration for the manufacture of such materials would be 3D printing (also known as additive manufacturing), enabling the precisely controlled design and production of 3-DOM ceria microstructures. There appear to be no reports to date of 3D printed ceria ceramics, although calcium silicate bioceramic bone scaffolds have been 3D printed with additions of up to 15 mol% CeO_2_ (Zhu et al., 2016). Porous reactive Al_2_O_3_ materials for syngas reforming were made by 3D printing (Fan et al., 2017), as was a 10 mol% ceria-stabilized zirconia/alumina composite (10CeTZP-Al2O3), robocast as a self-supporting circular lattice with interconnected 200–600 μm cells, giving a porosity of 30–50% (Goyos-Balla et al., 2017). 3D printing of ceramics is becoming ever more advanced, usually based on the extrusion of a precursor paste through needles as small as tens of μm in diameter, a process also known as robocasting. The green body that is produced can then be fired/sintered to produce the ceramic piece. Extremely complex porous structures can be produced, as shown in Figure 24a, and with resolution of 100 s or even tens of microns possible, porous ceria structures on this scale could be designed and manufactured. Robocasting is commonly used to produce bioceramic bone scaffolds, which have a porous microstructure like that shown in Figure 24b, where the ceramic and the voids both typically have diameters in the order of hundreds of microns. As an example of what is achievable, optical and SEM images of robocast and sintered Si-Ca-Na-P-O bioglass scaffolds produced by one of the authors (Ben-Arfa et al., 2019) are shown in Figures 24C–E.

In the case of the cork based biomimetic ceria developed by the authors, and discussed above, the processing of these cork-derived ceria ecoceramics needs to be optimized to fully replicate the cork structure, and we have begun this through experiments with both low and high pressure infiltration regimes of the pyrolysed cork precursors with ceria precursor solutions (Pereira, 2007). Recent results have shown that lower infiltration pressures led to higher porosity ceria ecoceramics made from cork templates. Such materials must have high surface areas based upon an interconnected pore network, which must be retained over many thousands of cycles and hours of operation at temperatures in excess of 1,400°C. There are many other suitable natural templates for biomorphic thermochemical materials which could be explored in the future as well.

One of the great advantages of cork is the fact that it is totally sustainable/renewable, and cork wastes were valorized in the production of the ceria ecoceramics. Other waste streams should also be investigated for the creation of renewable fuels from CO_2_. One example that has been investigated is red mud, a major waste product of the aluminum industry, which is produced in the order of millions of tons each year. Red mud is an iron rich, but highly alkaline, waste, which was used to make a catalyst for the conversion and storage of petroleum gas, a mixture of methane and other hydrocarbons produced offshore petroleum exploration. The iron-rich oxides were used to split CH_4_ to form CO/CO_2_ and water, with the resulting reduced metals further reacting to produce hydrogen and syngas (Teixeira et al., 2014). Such wastes have the potential to be useful as precursors for thermochemical reactive materials as well.

## Author Contributions

All authors contributed to the writing of this review.

### Conflict of Interest Statement

The authors declare that the research was conducted in the absence of any commercial or financial relationships that could be construed as a potential conflict of interest.

## References

[B1] Abanades S. Flamant G. (2006). Thermochemical hydrogen production from a two-step solar-driven water-splitting cycle based on cerium oxides. Solar Energy 80, 1611–1623. 10.1016/j.solener.2005.12.005

[B2] Ackermann S. Sauvin L. Castiglioni R. Rupp J. L. Scheffe J. R. Steinfeld A. (2015). Kinetics of CO_2_ reduction over nonstoichiometric ceria. J. Phys. Chem. C. 119, 16452–16461. 10.1021/acs.jpcc.5b03464 26693270 PMC4682555

[B3] Ackermann S. Scheffe J. R. Duss J. Steinfeld A. (2014b). Morphological characterization and effective thermal conductivity of dual-scale reticulated porous structures. Materials 7, 7173–7195. 10.3390/ma7117173 28788240 PMC5512629

[B4] Ackermann S. Scheffe J. R. Steinfeld A. (2014a). Diffusion of oxygen in ceria at elevated temperatures and its application to H_2_O/CO_2_ splitting thermochemical redox cycles. J. Phys. Chem. C. 118, 5216–5225. 10.1021/jp500755t

[B5] Ackermann S. Takacs M. Scheffe J. Steinfeld A. (2017). Reticulated porous ceria undergoing thermochemical reduction with high-flux irradiation. Int. J. Heat Mass Transf. 107, 439–449. 10.1016/j.ijheatmasstransfer.2016.11.032

[B6] APCOR (2016). Yearbook 2016, Portuguese Cork Association (Portugal). Available online at: https://www.apcor.pt/ (accessed November 10, 2018).

[B7] Azmi A. A. Ngadi N. Kamaruddin M. J. Zakaria Z. Y. Teh L. P. Annuar N. H. R. (2019). Rapid one pot synthesis of mesoporous ceria nanoparticles by sol-gel method for enhanced CO_2_ capture. Chem. Eng. Trans. 72, 403–408. 10.3303/CET1972068

[B8] Bader R. Venstrom L. J. Davidson J. H. Lipin'ski W. (2013). Thermodynamic analysis of isothermal redox cycling of ceria for solar fuel production. Energy Fuels 27, 5533–5544. 10.1021/ef400132d

[B9] Ben-Arfa B. A. E. Neto A. S. Palamá I. E. Miranda Salvado I. M. Pullar R. C. Ferreira J. M. F. (2019). Robocasting of ceramic glass scaffolds: sol-gel glass, new horizons. J. Euro. Ceram. Soc. 39, 1625–1634. 10.1016/j.jeurceramsoc.2018.11.019

[B10] Bhosale R. R. Takalkar G. Sutar P. Kumar A. Al Momani F. Khraisheh M. (2019). A decade of ceria based solar thermochemical H_2_O/CO_2_ splitting cycle. Int. J. Hydro. Energy 44, 34–60. 10.1016/j.ijhydene.2018.04.080

[B11] Bonk A. Maier A. C. Schlupp M. V. F. Burnat D. Remhof A. Delmelle R. (2015). The effect of dopants on the redox performance, microstructure and phase formation of ceria. J. Pow. Sources 300, 261–271. 10.1016/j.jpowsour.2015.09.073

[B12] Bulfin B. Lowe A. J. Keogh K. A. Murphy B. E. Lübben O. Krasnikov S. A. (2013). Analytical model of CeO_2_ oxidation and reduction. J. Phys. Chem. C. 117, 24129–24137. 10.1021/jp406578z

[B13] Carrillo R. J. Scheffe J. R. (2017). Advances and trends in redox materials for solar thermochemical fuel production. Solar Energy 156, 3–20. 10.1016/j.solener.2017.05.032

[B14] Centi G. Cum G. J. Fierro L. G. Lopez Nieto J. M. (2008). Direct Conversion of Methane, Ethane and Carbon Dioxide to Fuels and Chemicals. CAP Report, The Catalyst Group Resources. Available online at: https://www.yumpu.com/en/document/read/13122918/direct-conversion-of-methane-ethane-and-carbon-dioxide-to-fuels- (accessed August 5, 2019).

[B15] Centi G. Perathoner S. (2009). Opportunities and prospects in the chemical recycling of carbon dioxide to fuels. Catalysis Today 148, 191–205. 10.1016/j.cattod.2009.07.075

[B16] Cho H. Kodama T. Gokon N. Kim J. Lee S. Kang Y. (2017). Development and experimental study for hydrogen production from the thermochemical two-step water splitting cycles with a CeO_2_ coated new foam device design using solar furnace system. AIP Conf. Proc. 1850:100003 10.1063/1.4984460

[B17] Chuayboon S. Abanades S. Rodat S. (2019). Syngas production via solar-driven chemical looping methane reforming from redox cycling of ceria porous foam in a volumetric solar reactor. Chem. Eng. J. 356, 756–770. 10.1016/j.cej.2018.09.072

[B18] Chueh W. C. Falter C. Abbott M. Scipio D. Furler P. Haile S. M.. (2010). High-flux solar-driven thermochemical dissociation of CO_2_ and H_2_O using nonstoichiometric ceria. Science 330, 1797–1801. 10.1126/science.1197834 21205663

[B19] Chueh W. C. Haile S. M. (2010). A thermochemical study of ceria: exploiting an old material for new modes of energy conversion and CO_2_ mitigation. Phil. Trans. R. Soc. A. 368, 3269–3294. 10.1098/rsta.2010.0114 20566511

[B20] Djurovic D. Zinkevich M. Boskovic S. Strot B. Aldinger F. (2007). Densification behaviour of nano-sized CeO_2_. Mater. Sci. Forum. 555, 189–194. 10.4028/www.scientific.net/MSF.555.189

[B21] Fan N. C. Chen Y. Y. Chen K. Y. Wei W. C. Liu B. H. Wang A. B. (2017). Porous Al2O3 catalyst carrier by 3D additive manufacturing for syngas reforming. J. Ceram. Process. Res. 18, 676–682. Available online at: http://jcpr.kbs-lab.co.kr/file/JCPR_vol.18_2017/JCPR18-9/09.2017-164_676-682.pdf

[B22] Fornasiero P. Balducci G. Di Monte R. Kašpar J. Sergo V. Gubitosa G. (1996). Modification of the redox behaviour of CeO_2_ induced by structural doping with ZrO_2_?. J. Catal. 164, 173–183. 10.1006/jcat.1996.0373

[B23] Furler P. Scheffe J. Gorbar M. Moes L. Vogt U. Steinfeld A. (2012a). Solar thermochemical CO_2_ splitting utilizing a reticulated porous ceria redox system. Energy Fuels 26, 7051–7059. 10.1021/ef3013757

[B24] Furler P. Scheffe J. Marxer D. Gorbar M. Bonk A. Vogt U.. (2014). Thermochemical CO_2_ splitting via redox cycling of ceria reticulated foam structures with dual-scale porosities. Phys. Chem. Chem. Phys. 16, 10503–10511. 10.1039/C4CP01172D 24736455

[B25] Furler P. Scheffe J. R. Steinfeld A. (2012b). Syngas production by simultaneous splitting of H_2_O and CO_2_ via ceria redox reactions in a high-temperature solar reactor, powered by a high flux solar simulator Energy Environ. Sci. 5, 6098–6103. 10.1039/C1EE02620H

[B26] Ganesan K. Randrianalisoa J. Lipinski W. (2013). Effect of morphology on spectral radiative properties of three-dimensionally ordered macroporous ceria packed bed. J. Heat Transfer. 135:122701 10.1115/1.4024942

[B27] Gibbons W. T. Venstrom L. J. De Smith R. M. Davidson J. H. Jackson G. S. (2014). Ceria-based electrospun fibers for renewable fuel production via two-step thermal redox cycles for carbon dioxide splitting. Phys. Chem. Chem. Phys. 16, 14271–14280. 10.1039/C4CP01974A 24914875

[B28] Gil L. (2014). Cork: a strategic material. Front. Chem. 2:16. 10.3389/fchem.2014.00016 24790984 PMC3990040

[B29] Gladen A. C. Davidson J. H. (2016). The morphological stability and fuel production of commercial fibrous ceria particles for solar thermochemical redox cycling. Solar Energy 139, 524–532. 10.1016/j.solener.2016.10.029

[B30] Global CSS Institute (2018). The Global Status of CSS. Available online at: https://www.globalccsinstitute.com/resources/global-status-report/; https://indd.adobe.com/view/2dab1be7-edd0-447d-b020-06242ea2cf3b (accessed August 5, 2019).

[B31] Goyos-Balla L. García-Tuñón E. Fernández-García E. Díaz R. Fernández A. Prado C. (2017). Mechanical and biological evaluation of 3D printed 10CeTZP-Al_2_O_3_ structures. J. Euro. Ceram. Soc. 37, 3151–3158. 10.1016/j.jeurceramsoc.2017.03.012

[B32] Graves C. Ebbesen S. D. Mogensen M. Lackner K. S. (2011). Sustainable hydrocarbon fuels by recycling CO_2_ and H_2_O with renewable or nuclear energy. Renew. Sustain. Energy Rev. 15, 1–23. 10.1016/j.rser.2010.07.014

[B33] Haussener S. Steinfeld A. (2010). Effective heat and mass transport properties of anisotropic porous ceria for solar thermochemical fuel generation, in Conference Proceedings 2010 AIChE Annual Meeting, 10AIChE (Salt Lake City, UT). 10.3390/ma5010192 PMC5448949 28817039

[B34] Haussener S. Steinfeld A. (2012). Effective heat and mass transport properties of anisotropic porous ceria for solar thermochemical fuel generation. Materials 5, 192–209. 10.3390/ma5010192 28817039 PMC5448949

[B35] Holmen A. (2009). Direct conversion of methane to fuels and chemicals. Catal. Today 142, 2–8. 10.1016/j.cattod.2009.01.004

[B36] Inaba H. Nakajima T. Tagawa H. (1998). Sintering behaviors of ceria and gadolinia doped ceria. Solid State Ionics 106, 263–268. 10.1016/S0167-2738(97)00496-7

[B37] International Energy Agency (IEA) (2008). World Energy Outlook. Paris: International Energy Agency.

[B38] Keene D. J. Lipinski W. Davidson J. H. (2014). The effects of morphology on the thermal reduction of nonstoichiometric ceria. Chem. Eng. Sci. 111, 231–243. 10.1016/j.ces.2014.01.010

[B39] Knoblauch N. Dörrer L. Fielitz P. Schmücker M. Borchardt G. (2015). Surface controlled reduction kinetics of nominally undoped polycrystalline CeO_2_. Phys. Chem. Chem. Phys. 17, 5849–5860. 10.1039/C4CP05742B 25630597

[B40] Koepf E. Alxneit I. Wieckert C. Meier A. (2017). A review of high temperature solar driven reactor technology: 25 years of experience in research and development at the Paul Scherrer Institute. Appl. Energy. 188, 620–651. 10.1016/j.apenergy.2016.11.088

[B41] Kovacevic M. Mojet B. L. van Ommen J. G. Lefferts L. (2016). Effects of morphology of cerium oxide catalysts for reverse water gas shift reaction. Catal. Lett. 146, 770–777. 10.1007/s10562-016-1697-6

[B42] Lange M. Roeb M. Sattler C. Pitz-Paal R. (2015). Efficiency assessment of a two-step thermochemical water-splitting process based on a dynamic process model. Int. J. Hydrogen Energy. 40, 12108–12119. 10.1016/j.ijhydene.2015.07.056

[B43] Lanzafame P. Abate S. Ampelli C. Genovese C. Passalacqua R. Centi G.. (2017). Beyond solar fuels: renewable energy-driven chemistry. Chem. Sus. Chem. 10, 4409–4419. 10.1002/cssc.201701507 29121439

[B44] Le Gal A. Abanades S. Flamant G. (2011). CO_2_ and H_2_O splitting for thermochemical production of solar fuels using nonstoichiometric ceria and ceria/zirconia solid solutions. Energy Fuels 25, 4836–4845. 10.1021/ef200972r

[B45] Le Quéré C. Andrew R. M. Friedlingstein P. Sitch S. Hauck J. Pongratz J. (2018). Global Carbon Budget 2018. Earth Syst. Sci. Data 10, 2141–2194. 10.5194/essd-10-2141-2018

[B46] Lorentzou S. Karagiannakis G. Dimitrakis D. Pagkoura C. Zygogianni A. Konstandopoulos A. G. (2015). Thermochemical redox cycles over Ce-based oxides. Energy Procedia. 69, 1800–1809. 10.1016/j.egypro.2015.03.152

[B47] Malonzo C. D. De Smith R. M. Rudisill S. G. Petkovich N. D. Davidson J. H. Stein A. (2014). Wood-templated CeO_2_ as active material for thermochemical CO production. J. Phys. Chem. C. 118, 26172–26181. 10.1021/jp5083449

[B48] Marxer D. Furler P. Scheffe J. Geerlings H. Falter C. Batteiger V. (2015). Demonstration of the entire production chain to renewable kerosene via solar thermochemical splitting of H_2_O and CO_2_. Energy Fuels 29, 3241–3250. 10.1021/acs.energyfuels.5b00351

[B49] Marxer D. Furler P. Takacs M. Steinfeld A. (2017). Solar thermochemical splitting of CO2 into separate streams of CO and O_2_ with high selectivity, stability, conversion, and efficiency. Energy Environ. Sci. 10, 1142–1149. 10.1039/C6EE03776C

[B50] Mogensen M. Sammes N. M. Tompsett G. A. (2000). Physical, chemical and electrochemical properties of pure and doped ceria. Solid State Ionics. 129, 63–94. 10.1016/S0167-2738(99)00318-5

[B51] Novais R. M. Pullar R. C. (2019). Comparison of low and high pressure infiltration regimes on the density and highly porous microstructure of ceria ecoceramics made from sustainable cork templates. J. Euro. Ceram. Soc. 39, 1287–1296. 10.1016/j.jeurceramsoc.2018.11.050

[B52] Oliveira F. A. C. Barreiros M. A. Abanades S. Caetano A. P. F. Novais R. M. Pullar R. C. (2018). Solar thermochemical CO_2_ splitting using cork-templated ceria ecoceramics. J. CO_2_ Util. 26, 552–563. 10.1016/j.jcou.2018.06.015

[B53] Pereira H. (2007). Cork: Biology, Production and Uses, 1st Edn. Amsterdam: Elsevier Science B.V 10.1016/B978-044452967-1/50013-3

[B54] Pullar R. C. Gil L. Oliveira F. A. C. (2016). Biomimetic cork-based CeO_2_ ecoceramics for hydrogen generation using concentrated solar energy. Ciência Tecnologia dos Mater. 28, 23–28. 10.1016/j.ctmat.2016.04.002

[B55] Pullar R. C. Marques P. Amaral J. Labrincha J. A. (2015). Magnetic wood-based biomorphic Sr_3_Co_2_Fe_24_O_41_ Z-type hexaferrite ecoceramics made from cork templates. Mater. Design. 82, 297–303. 10.1016/j.matdes.2015.03.047

[B56] Pullar R. C. Novais R. M. (2017). Cork-based biomimetic ceramic 3-DOM foams. Mater. Today 20, 45–46. 10.1016/j.mattod.2016.12.004

[B57] Rhodes N. R. Bobek M. M. Allen K. M. Hahn D. W. (2015). Investigation of long term reactive stability of ceria for use in solar thermochemical cycles. Energy 89, 924–931. 10.1016/j.energy.2015.06.041

[B58] Roeb M. Steinfeld A. Borchardt G. Feldmann C. Schmücker M. Sattler C. (2016). Solar syngas: Results from a virtual institute developing materials and key components for solar thermochemical fuel production. AIP Conf. Proc. 1734:120007 10.1063/1.4949209

[B59] Rudisill S. G. Venstrom L. J. Petkovich N. D. Quan T. Hein N. Boman D. B. (2013). Enhanced oxidation kinetics in thermochemical cycling of CeO_2_ through templated porosity. J. Phys. Chem. C. 117, 1692–1700. 10.1021/jp309247c

[B60] Scheffe J. R. Jacot R. Patzke G. R. Steinfeld A. (2013). Synthesis, characterization, and thermochemical redox performance of Hf^4+^, Zr^4+^, and Sc^3+^ doped ceria for splitting CO_2_. J. Phys. Chem. C. 117, 24104–24114. 10.1021/jp4050572

[B61] Scheffe J. R. Welte M. Steinfeld A. (2014). Thermal reduction of ceria within an aerosol reactor for H_2_O and CO_2_ splitting. Ind. Eng. Chem. Res. 53, 2175–2182. 10.1021/ie402620k

[B62] Siegel N. P. Miller J. E. Ermanoski I. Diver R. B. Stechel E. B. (2013). Factors affecting the efficiency of solar driven metal oxide thermochemical cycles. Ind. Eng. Chem. Res. 52, 3276–3286. 10.1021/ie400193q

[B63] Singh M. Martínez-Fernández J. Arellano-López A. R. (2003). Environmentally conscious ceramics (ecoceramics) from natural wood precursors. Curr. Opin. Solid State Mater. Sci. 7, 247–254. 10.1016/j.cossms.2003.09.004

[B64] Takacs M. Ackermann S. Bonk A. Neises-von Puttkamer M. Haueter P. Scheffe J. R. (2017). Splitting CO_2_ with a ceria-based redox cycle in a solar-driven thermogravimetric analyser. AIChE J. 63, 1263–1271. 10.1002/aic.15501 28405030 PMC5367271

[B65] Takacs M. Hoes M. Caduff M. Cooper T. Scheffe J. R. Steinfeld A. (2016). Oxygen nonstoichiometry, defect equilibria, and thermodynamic characterization of LaMnO_3_ perovskites with Ca/Sr A-site and Al B-site doping. Acta Mater. 103, 700–710. 10.1016/j.actamat.2015.10.026

[B66] Teixeira I. F. Medeiros T. P. V. Freitas P. E. Rosmaninho M. G. Ardisson J. D. Lago R. M. (2014). Carbon deposition and oxidation using the waste red mud: a route to store, transport and use offshore gas lost in petroleum exploration. Fuel 124, 7–13. 10.1016/j.fuel.2014.01.088

[B67] Venstrom L. J. (2012). The Oxidation of Zinc Vapor and Non-stoichiometric Ceria by Water and Carbon Dioxide to Produce Hydrogen and Carbon Monoxide. Ph.D. Thesis, Minnesota, MN, University of Minnesota.

[B68] Venstrom L. J. Petkovich N. Rudisill S. Stein A. Davidson J. H. (2011). The oxidation of macroporous cerium and cerium-zirconium oxide for the solar thermochemical production of fuels, in Proceedings of ASME 2011 5th International Conference on Energy Sustainability (Washington, DC), 1585–1593. 10.1115/ES2011-54160

[B69] Venstrom L. J. Petkovich N. Rudisill S. Stein A. Davidson J. H. (2012). The effects of morphology on the oxidation of ceria by water and carbon dioxide. J. Solar Energy Eng. 134:011005 10.1115/1.4005119

[B70] Wheeler V. M. Randrianalisoa J. Tamma K. K. Lipinski W. (2014). Spectral radiative properties of three-dimensionally ordered macroporous ceria particles. J. Quant. Spectr. Radiative Transf. 143, 63–72. 10.1016/j.jqsrt.2013.08.007 24663356

[B71] Zhu L. Lu Y. Li F. (2018). Reactivity of Ni, Cr and Zr doped ceria in CO_2_ splitting for CO production via two-step thermochemical cycle. Int. J. Hydro. Energy. 43, 13754–13763. 10.1016/j.ijhydene.2018.02.015

[B72] Zhu M. Zhang J. Zhao S. Zhu Y. (2016). Three-dimensional printing of cerium-incorporated mesoporous calcium-silicate scaffolds for bone repair. J. Mater. Sci. 51, 836–844. 10.1007/s10853-015-9406-1

